# Supplementary material for: Impact evaluation of a digital health platform empowering Kenyan women across the pregnancy-postpartum care continuum: A cluster randomized controlled trial
Source: PLoS Med. 2025 Feb 3;22(2):e1004527. doi: 10.1371/journal.pmed.1004527 (PMC11835334; doi:10.1371/journal.pmed.1004527)
Supplement: S1 Protocol — (PDF) [file pmed.1004527.s001.pdf]

Instructions: Complete this template to provide IRB members and designated reviewers with sufficient information to conduct a substantive review of human research. If applicable, submit a Sponsor's Protocol in addition to this document. Detailed instructions for preparing this template can be found in the Investigator's Manual. If the proposed human research is eligible for an Exemption Determination, see Appendix H of the Investigator Manual.

**General Information**	
**Protocol/ESTR Record Number** (if assigned):	
**Version Number: 1.0**	**Version Date: 8/5/2021**
**Principal Investigator (PI):** Jessica Cohen, PhD; Margaret McConnell, PhD	
**Principal Investigator's Harvard Affiliation: Faculty**	
**Study Implementation Leader** (if different from above-named PI):	
**Name of Person(s) Completing This Form**: Wei Chang, PhD	
**Protocol Title:** Evaluating a package intervention to improve quality of maternal and neonatal care in Kenyan public health hospitals	

1. Specific Aims

This protocol describes a study to conduct an evaluation of a package of maternal and neonatal health programs being implemented in Kenya by Jacaranda Health. The evaluation will be a randomized controlled trial (RCT) to assess the effects of a package intervention on patient and provider outcomes. The evaluation is a collaboration between the PI team (who are leading the research design and evaluation), Jacaranda Health and Innovations for Poverty Action (Kenya).

The aims of our study are as follows:

1. To assess the intervention's impact on patient health care utilization, knowledge, and health outcomes.
2. To assess the intervention's impact on quality of maternal and neonatal care and health care provider knowledge.
3. To assess health facilities' readiness to provide maternal and neonatal care.
4. To assess the implementation and sustainability of the intervention.

2. Background and Significance

2.1 Provide the scientific background and rationale for the research.

Reducing maternal and newborn mortality and morbidity remains a major public health priority in many countries in sub-Saharan Africa (SSA). In Kenya, maternal mortality and neonatal mortality remain high at 3.4 and 21 per 1,000 live births respectively.[1,2] A recent study suggests that more than half of the avertable maternal and neonatal deaths in low- and middle-income countries could be amenable to better quality care,[3] but two key challenges limit health systems' ability to improve quality of care and patient health outcomes in Kenya.

The first challenge is that providers do not always deliver high-quality care that could avert or mitigate life-threatening pregnancy and postpartum complications due to lack of knowledge and skills.[4] Research has shown that training in emergency obstetric care is effective in improving some maternal

and neonatal outcomes, but one-off dissemination of guidelines is often insufficient to sustain improvement in providers' knowledge or practice in low-resource settings.[5,6] Mentorship programs that provide hands-on and longer-term support to health workers aim to achieve sustained improvements in quality of care through developing health workers' skills and competencies. However, evaluations of their effectiveness often focus on provider knowledge or practice alone without assessing health outcomes and very few studies are conducted in SSA using experimental designs.[7–9]

The second challenge is that pregnant women and mothers are often not empowered or informed to seek care at the appropriate time, which is often referred to as the First Delay in the Three Delays Model that examines barriers to obstetric care in low- and middle-income countries (LMIC).[10] This delay in the decision to seek care has direct consequences on the survival of mother and baby and has been associated with women's knowledge of maternal and neonatal danger signs.[11,12] Such delays in care-seeking could happen along multiple points in the pregnancy and postpartum continuum. Pregnant women often do not have adequate support during pregnancy as 62% of women in Kenya do not achieve 4 or more antenatal care visits, creating missed opportunities for catching signs of complications ahead of time.[13] In the critical 6–8-week postpartum window, 43% of women in Kenya do not get a postnatal care checkup for their health.[13] To address such delays, mobile health interventions have become increasingly popular in LMIC to provide information, encourage care-seeking, and provide support to pregnant women and mothers, but multiple systematic reviews suggest that mHealth interventions' impact on maternal and neonatal outcomes is inconclusive due to poor methodological quality and lack of studies that evaluate patient outcomes.[14–16]

Given these challenges in improving quality of care, the limitations of stand-alone interventions, and the lack of rigorous evaluations, we are embarking on an impact evaluation to assess the effects of a package intervention that addresses both provider- and patient-side barriers to achieving better maternal and neonatal health outcomes.

2.2 Describe the significance of the research, and how it will contribute to generalizable knowledge. *(Generalizable: Universally or widely applicable. Knowledge: Truths, facts, information.)*

The subject of the planned impact evaluation is a package of two complementary, low-cost solutions that address drivers of poor maternal and neonatal health (MNH) outcomes in Kenya developed by our in-country partner Jacaranda Health. The first - "MENTORS" - is a facility-based program that efficiently increases and sustains provider skills in basic and emergency obstetric and newborn care. The second is "PROMPTS", a digital health platform for mothers that aims to increase care-seeking behavior at the right time and place. These interventions are designed to address the quality challenges described above: MENTORS aims to equip providers with the skills to handle general and emergency obstetric situations and support providers in consistently implementing those skills, and PROMPTS aims to inform and empower pregnant women and new mothers to seek care at the right time and place. This solution package has been designed for integration in Kenyan public hospitals and demand for expansion is increasing. However, these interventions have not yet been subject to rigorous impact evaluation.

This study aims to assess the impact of the package intervention on quality of care and MNH outcomes. The findings from this study will contribute to the evidence on 1) what facility, provider, patient factors would affect quality of care and MNH outcomes and 2) the effectiveness of m-Health and training interventions in similar settings. We plan to publish the study findings in research journals.

3. Research Locations and Collaborating Sites

Research Locations refer to the geographic location that the research will take place, not to the institutions or researchers you may be collaborating with. All Research Locations should be listed in ESTR as a Research Location. Collaborating Sites refer to institutions or researchers that are also taking part in the research study. All Collaborating Sites should be listed in ESTR as a Participating Site.

3.1. Where will the research activities take place? (check all that apply)

☒	**At Harvard;** *list any non-Harvard Longwood Medical Area (LMA) Schools here: A list of all Harvard Schools can be found here.*
☐	**At another location in Massachusetts;** *specify here:*
☐	**In another state in the U.S.;** *specify here:*
☒	**Internationally;** *specify here: Kenya*
☐	**Remotely;** *specify here if any remote participant will be located in the European Economic Area (EEA), European Union (EU), and/or United Kingdom (UK) while participating:*

3.2. Describe the sites or locations where the research will be conducted or overseen by the Harvard PI. (If conducting the study virtually or remotely, indicate the location of the researcher who is conducting the study.)

The study sites are about 40 public or faith-based hospitals and health centers that provide maternity care services located in eight to ten counties in Kenya. The Harvard PIs will design and provide oversight for the research with close collaboration from Jacaranda Health; the field work will be carried out by the local implementation partner at Innovations for Poverty Action (Kenya).

3.3. Describe plans for communication among sites regarding adverse events, interim results, protocol modifications, monitoring of data, etc. ☐ N/A.

This study is a collaboration between the Harvard T. H. Chan School of Public Health, Jacaranda Health (JH), a non-profit organization in Nairobi, Kenya, and Innovations for Poverty Action (IPA), a research nonprofit organization that serves as the study's implementing partner. Investigators and research management from Harvard and Jacaranda will hold regular management-level meetings/calls together with the IPA Research Manager and Research Associate to keep up to date on study progress and to share information across collaborating sites of unexpected negative outcomes, interim results analysis planning, protocol modifications,

monitoring of data, etc. In-person oversight from the IPA management team will be ongoing throughout the study. Additionally, there will be opportunities for enumeration staff to have in-person group feedback sessions. The Research Manager and Research Associate at IPA will stay in frequent communication with the investigators about any issues related to data quality, adverse events and protocol modifications. Any issues will be reported to the investigators within 24 hours.

3.4. Describe any local (international or state) laws, regulations, and/or customs affecting the research (e.g., age of majority, mandatory reporting requirements, etc). ☐ N/A.

Oversight of research in Kenya involving human subjects requires two levels of approval: (1) Approval is required at the organizational level by Institutional Ethics and Review Committees (IERCs) and (2) section 17 (1) of the Science, Technology and Innovation Act, 2013 requires all persons intending to undertake research in Kenya to apply to the National Commission for Science, Technology and Innovations (NACOSTI) for the grant of a Research License.

Those under 18 years of age (but at least 15 years of age) who are married, a mother, pregnant, or a household head are considered a mature minor in Kenya. Mature minors are considered adults in the study setting and can consent on their own.

3.5. Identify any approvals or permissions required of collaborating institutions, community leaders, or government officials, including approval from another IRB or local research ethics committee. *Upload copies to the "Study-Related Documents" page in ESTR.* **☐ N/A.**

Research ethics approval for the study with annual reviews will be obtained from the Scientific Ethics Review Unit at AMREF (Kenya) in addition to Harvard School of Public Health.

County government approval: Prior to beginning work in each county, an approval letter will be signed by the respective county health directors. The letter is signed after the in-country study team presents the study protocol to members of the county health management and research team.

Facility approval: Prior to beginning the study, each facility in-charge will review and approve the study. Letter of approval will be signed by respective hospital medical superintendent.

3.6. Will you collaborate with any researchers not affiliated with Harvard to carry out this study?
☐ No ☒ Yes: *If yes, list which institutions they are affiliated with. If they are not affiliated with an institution, indicate that here. If yes, also indicate their responsibilities and scope of work in conducting this research.*

Collaborating researchers are affiliated with Jacaranda Health and IPA (Kenya) including:
- Cynthia Kahumbura (Jacaranda Health): her scope of work includes leading Jacaranda's programs, and ensuring the operational elements of the study and interventions are adequately resourced.

- Sharon Akinyi (Jacaranda Health): her scope of work includes to lead county introduction, oversee overall study implementation from Jacaranda's side, and ensure the intervention launches at the right times.
- Ginger Golub & John Mungai (IPA): they will provide oversight of the data collection activities to ensure compliance with the study protocol and to ensure integrity of data quality. They will supervise the Research Associate (Catherine Gakii) at IPA and will ensure the project stays within the agreed upon timeline, field plan, and budget.
- Catherine Gakii (IPA): her scope of work includes managing data collection activities and ensuring adherence to the protocol. She will also conduct data quality assurance checks throughout data collection.
- Violet Naanyu (co-investigator based in Kenya): she will lead a qualitative evaluation of the MENTORS program, including qualitative study design, data collection, and analyses. She will also advise on other parts of the RCT for mixed-methods analyses.

3.7. Will your collaborators interact with human subjects, have access to identifiable data/specimens, and/or be responsible for the design, conduct, oversight, or reporting of the research?
☐ **No** ☒ **Yes:** *If yes, indicate if the collaborators will obtain their own IRB review.*

The study will be reviewed by a local IRB in Kenya (AMREF). Collaborators based in Kenya will be covered by the local IRB.

3.8. Will any institution conducting research activities as part of this study, including collaborators, rely on Harvard LMA for IRB review?
☒ **No** ☐ **Yes:** *If yes, list each relying institutions, their site responsible Investigator, and describe what research activities will be conducted there.*

4. Study Team

4.1. Describe the scope of work of the Harvard PI and research team. Indicate who is responsible for the design, conduct, implementation, and/or reporting of the research. Indicate who is responsible for the creation, design, and/or implementation of the study documents/tools.

	Jessica Cohen (Co-PI)	Margaret McConnell (Co-PI)	Wei Chang (Postdoctoral Fellow)
Study design	X	X	X
Implementation	X		
Research reporting	X	X	X
Creation, design, and implementation of study documents/tools	X	X	X

4.2. Describe the Principal Investigator's experience conducting research at the study site(s) and familiarity with the local research context.

Jessica Cohen, PhD is Associate Professor of Global Health at the Harvard School of Public Health and Affiliated Professor at the Jameel-Poverty Action Lab (J-PAL). Dr. Cohen received her bachelor's degree in Economics from Wesleyan University and was a National Science Foundation Graduate Research Fellow at MIT, where she received her doctorate in economics. Her research applies the methods of program design, randomized trials, and impact evaluation to maternal and child health programs and policies in sub-Saharan Africa. She has conducted many previous randomized controlled trials on maternal and child health issues in East Africa, including several studies in Kenya.

Margaret McConnell, PhD is Associate Professor of Global Health Economics at the Harvard Chan School of Public Health. Dr McConnell received a bachelor's degree in social sciences from Wesleyan University and a PhD in social sciences from the California Institute of Technology. Her research applies tools from economics and behavioral sciences to understand strategies to improve to health outcomes for marginalized populations, with a particular focus on behavioral economic theory, experimental methods and impact evaluation strategies. She has been principal investigator or co-principal investigator on more than five randomized control trials, including two trials in Kenya.

4.3. Describe how the Principal Investigator will ensure that sufficient time is devoted to conducting and completing the research.

All of the principal investigators on this grant have adequate funding in the grant for their effort on the project. They are also working with a highly skilled postdoctoral fellow, Research Manager and administrative staff to conduct the study.

4.4. Describe how all research staff members are trained to ensure that they are adequately informed about the protocol and study-related duties.

Wei Chang, PhD is a Postdoctoral Research Fellow at Harvard Chan School of Public Health. She received master's degrees in public health and social work from Washington University in St. Louis and a PhD in health policy and management from University of North Carolina at Chapel Hill. Wei conducts cross-disciplinarily population research and evaluates interventions in maternal, sexual, and reproductive health in low-resource settings. She has participated in several randomized controlled trials in low- and middle-income countries, including three studies on HIV in East Africa and one study on maternal and newborn health in rural China.

4.5. Describe the minimum qualifications for each research role (e.g., RN, social worker, phlebotomist, statistician), their experience in conducting research, and their knowledge of the local research context.

All data collection staff will be employed by our implementation partner IPA. The following staff (and staff with similar roles) will be employed by IPA:

Research Associate:

- Master's degree in public health, social sciences, public policy, or related fields is desirable.
- Bachelor's degree in public health, social sciences, public policy, or related fields required.
- 4-6 research experience, in the field of maternal health, strongly preferred
- Prior work experience (>4 years) conducting field research in developing contexts
- Ability to manage and clean complex datasets, preferably, using STATA
- Ability to program complex surveys in SurveyCTO
- Excellent management and organizational skills
- Demonstrated ability to manage field teams and data collection at various levels (planning, piloting, collecting, transcription, backchecking, financial budgeting and reporting, human resources and team building)
- Flexible, self-motivating, able to manage multiple tasks efficiently, and team player
- Demonstrated ability to manage high-level relationships with partner organizations, PIs and policymakers
- Excellent communication skills with fluency in English and Swahili

Enumerators:

- Strong writing and analytical skills and able to work with minimum supervision.
- Experience working in research and field data collection
- Understanding and experience in collecting electronic data using tablets
- *Additional requirement for those who conduct delivery observations:* must be pursuing or graduating with a (1) Medical (2) KRCN Nursing or BscN Degree within the last 2 years, and experience in maternal and newborn care (medical, nursing and midwifery schools should be accredited by the relevant national bodies in Kenya.)

The enumerators will further receive specific in-depth training by IPA staff with clinical expertise on ethics and proper conduct expected while operating in a maternity ward. This will prepare them for how to react in various scenarios to maintain the highest ethical standards.

5. Study Design

5.1. Describe the study design type.

This study is designed as a parallel arm cluster randomized control trial (RCT). Randomization of the intervention package will occur at the facility level. Overall, 40 - 50 facilities across 8 - 10 counties will be randomized into either the intervention or control group. Facilities will be chosen to ensure sufficient geographical distance from all other facilities included in the sample.

Data will be collected at multiple points during the study period (see the table below). The baseline data collection period will last for about a month in each facility. Baseline data collection will include facility assessment, register data extraction, and health worker interviews. The MENTORS program will be rolled out in intervention facilities after the baseline data collection.

The facility-based endline data collection will occur 4-5 months after the end of baseline and will last for 1-2 months in each facility. The facility-based endline data collection will include register data extraction, health worker interviews, and observations of quality of care during labor and delivery.

Process evaluation includes extraction of administrative program data collected by Jacaranda Health to monitor intervention implementation. Jacaranda Health will share this data with us throughout the study period.

We will recruit pregnant women from antenatal care clinics during the baseline data collection period. For this sample, we will conduct two follow-up surveys by phone—one towards the end of pregnancy and another at around 7- to 8-weeks postpartum. Women enrolled from the intervention facilities will be offered the PROMPTS intervention immediately after the baseline survey.

After the endline, facilities in the control group will also receive the intervention package.

In summary, we plan to collect the following data:

	Facility-Based Data Collection			*Phone Surveys with Pregnant Women Cohort*	
	**Baseline**	**In-between**	**Endline**	**End of pregnancy**	**7- to 8-weeks postpartum**
Facility assessment	X				
Register data extraction	X		X		
Health worker interview	X		X		
Patient interview	X			X	X
Delivery observation			X		
Process evaluation*		X			

*Process evaluation will be conducted in intervention facilities only.

5.2. Does the study involve more than one participant group?
☐ **No** ☒ **Yes:** *If yes, identify each group here and throughout all applicable sections.*

I. Facility and maternity in-charges, pharmacists, phlebotomists, and other health workers that are familiar with the operations at different departments at selected health facilities
II. Nurses, midwives, and other health workers working in the maternity wards at the selected health facilities
III. Women visiting health facilities for antenatal care
IV. Women admitted to health facilities for labor and delivery

5.3. Indicate the total duration of a participant's involvement.

I. In-charges and other providers at health facilities will be engaged for approximately 2-3 days during the baseline data collection period for the facility assessment.

II. Nurses, midwives, and other health workers in the maternity wards will be engaged on an ongoing basis during the endline survey over a period of 1-2 months for delivery observations. Nurses and midwives will also be engaged for the provider survey, which will last about 1 hour during both baseline and endline data collection periods.

III. Women visiting health facilities for antenatal care will be engaged for about 10-15 minutes to complete a survey. They will be contacted by phone for the two follow-up surveys. These phone surveys will last for about 15-20 minutes. In addition to the phone calls for data collection, there is a small chance that participants will receive a follow-up call for data quality assurance.

IV. Women admitted to selected health facilities for labor and delivery will be engaged from arrival/admission to facility to end of discharge from facility in the endline data collection periods.

5.4. Indicate the total number of participants to be screened (if applicable) and/or enrolled (i.e., signed consent form). If the proposed research involves secondary data analyses only, indicate the number of data, documents, records, and/or specimens that will be obtained.

- We plan to enroll about 40 facilities for the facility assessment.
- We plan to enroll about 200 nurses and midwives (about 5 per facility) from study facilities for the provider survey.
- We plan to enroll about 4800 women (about 120 per facility) who are seeking antenatal care in study facilities.
- We plan to enroll about 4800 women admitted for labor and delivery (about 120 per facility) for delivery observations.
- We plan to collect register data for the time period that covers 12 months prior to the baseline and up to 12 months after the endline.

5.5. List inclusion and exclusion criteria and describe the screening process. Provide a rationale for any specific exclusion criteria.

I. Facilities are eligible for the study if they 1) have at least 50 normal vaginal deliveries per month on average; 2) have at most 400 normal vaginal deliveries per month on average; 3) are owned by the government or a faith-based organization; 4) have no potentially confounding ongoing research or quality-improvement programs at the time of selection (especially mentorship or mHealth programs).

II. Providers are eligible for the provider interview if they 1) are nurses or midwives; 2) provide maternity services; 3) have no plan to move to a different facility in the next 6 months (baseline only).

III. Pregnant women presenting for antenatal visits are eligible for the study if they 1) are at least 15 years old; 2) have access to a mobile phone; and (3) are at least 16 weeks pregnant or in Month 5, 6, 7, 8, and 9 of pregnancy.
Those under 18 years of age who are considered a mature minor will participate. Mature

minors are those who are married, a mother, pregnant, or a household head who are under 18 years of age.

IV. Patients admitted for labor and delivery at participating facilities are eligible for the study if they 1) are at least 15 years old; 2) are admitted for labor and delivery at the maternity ward.
Pregnant women are excluded from the study if they 1) are presenting for conditions other than labor and delivery (e.g., patients admitted for prenatal observation or complications from abortion), 2) are immediately transferred to another hospital. We apply these exclusion criteria because of the study's focus on evaluating the intervention's impact on maternal and neonatal care in selected facilities.

5.6. Check each age range applicable to participants or data/specimens.

☐ Ages 0-7
☒ Ages 8-17
☒ Ages 18+
☐ Other; specify:

5.7. Describe study procedures.

We will engage in six categories of data collection procedures: facility assessment; register data extraction; health worker interviews; patient interviews with a cohort of pregnant women recruited from antenatal care clinics; delivery observation; and process evaluation.

Facility assessment. Enumerators will conduct a facility assessment at the time of baseline. No individually identifiable information will be collected in the facility assessment.

1) At the start of data collection, the enumerator will identify who holds the position of facility In-Charge and Head Midwife and schedule appointments with them.
2) During this meeting, the enumerator will share the purpose and content of the facility audit and ask if health workers that are familiar with the facility operations are willing to meet for an interview. These health workers may include the In-Charge, Head Midwife, and pharmacists. Interviews will be scheduled during a time when these staff are not busy with other duties and have had enough time to update the register from the most current numbers.
3) The enumerator will meet designated staff at the scheduled time. The enumerator will identify a quiet and private space where the enumerator and staff member will sit together for the duration of the interview. The enumerator will ask the required information and ask the staff member any clarifying questions. Each respondent will be interviewed individually and privately and preferably outdoors. The interviews may be recorded for quality control purposes.

Register data extraction: Enumerators will capture data on delivery outcomes, complications, and incidence of morbidity and mortality from facility registers in consultation with facility in-charges and head midwives. Enumerators will complete the Register Data Form by copying the information from the facility registers (e.g., the MOH 333 Maternity Register) in the baseline and the endline.

1) In the baseline and the endline, enumerators will collect register data without any personally identifiable information (e.g., name, date of birth, phone number, etc.). Register data entered from the period that covers 12 months prior to the baseline and up to 12 months after the endline will be extracted.

Health worker interviews. Enumerators will interview about 2 - 8 health workers per facility at both the baseline and the endline:

1) The enumerator will approach all health workers that meet the inclusion criteria at the maternity ward. The enumerator will approach health workers individually, share with them the purpose of the interview, and ask whether they are willing to participate.
2) The enumerator will schedule an interview with each health worker at a time when the staff is not busy with other duties.
3) The enumerator will meet each health worker at the scheduled time. The enumerator will identify a quiet and private space where the enumerator and the health worker will sit together for the duration of the interview. The enumerator will ask the required information and ask the staff member any clarifying questions. Each respondent will be interviewed individually and privately, and preferably outdoors. The interviews may be recorded for quality control purposes.

Patient interviews with a cohort of pregnant women: we will recruit pregnant women who visit health facilities for antenatal care. One or two enumerators will be stationed outside of the antenatal care clinic in each facility to enroll prenatal patients. Pregnant women who consent to participate in the study will be contacted by phone toward the end of their pregnancy and at about 7 – 8 weeks postpartum for follow-up surveys. Women from intervention facilities who agree to receiving text messages will be enrolled in the PROMPTS program by Jacaranda Health. Procedures for data collection are as follows:

1) The enumerators will be on site before the antenatal care clinic is open to see patients.
2) Enumerators will approach women before or after they receive antenatal care (e.g., to wait for her providers or right after the completion of her visit) and recruit them to participate in the study. Enumerators will obtain women's consent and conduct a short survey on health care utilization, knowledge, and demographics (including women's contact information for follow-up calls). This will be done in a private space to protect participants' privacy and preferably outdoors. If the respondent does not have a mask, the enumerator will provide one.
3) Enumerators will also make a short announcement periodically to those in the waiting bay/queue that they are recruiting participants for a research study and can explain the details of the study for those interested outdoors.
4) Enumerators will conduct two short follow-up interviews by phone. The first interview will occur towards the end of pregnancy and the second interview will occur at around 7- to 8-week postpartum. These interviews will contain questions about women's health care utilization, health knowledge, self-efficacy, health outcomes, and experience with the intervention (if applicable). Enumerators will confirm the identity of the participant before proceeding with the phone interviews. Participants who complete surveys will receive a small SMS credit. The phone interviews may be recorded for quality control purposes.

Delivery observations: medically trained enumerators will observe labor, delivery, and postpartum care and record information on quality of care during the endline period.

1) Enumerators, with the aid of the maternity in-charge, will decide in advance where to sit in different parts of the maternity ward so as to be able to observe the actions of the health workers, but be out of the way and non-disruptive.
2) Introduction and consent:
    a. Upon arrival of a woman in labor, an enumerator will join the health staff in approaching and greeting the woman. Following the first examination and confirmation of admission, the enumerator will wait until an appropriate moment to begin consent procedures. The enumerator will not approach a woman for consent who is not stable or able to focus on the consent process. The enumerator will ask for the woman's consent before her first examination for her care to be observed and her records to be transcribed from the facility records (e.g., maternity register). The enumerator will not record information about the woman until consent has been given.
    b. In some situations, a woman may be unable to give consent upon arrival to the facility but may later be sufficiently stable to give consent. In this situation, an enumerator may approach the woman once she is stable and able to give consent. As above, the enumerator will not record information about the woman until consent has been given.
3) Collecting clinical quality observation data:
    a. If the woman consents to participate in the study, the enumerator will thank her and inform her that he/she and the other enumerators will not be giving any care to her during the duration of her delivery, but will only be present as observers.
    b. Enumerators will avoid speaking to patients or health workers, and will avoid sudden movements that may draw any attention.
    c. The enumerator will follow the staff and woman to the maternity ward.
    d. The enumerator will observe labor and delivery for as long as it is on-going, up until time of discharge from the facility, time when a patient is referred out and leaves the facility, or when a patient is going to receive a Cesarean section.
    e. The enumerator may use the patient chart, partograph, the woman's antenatal care book, bill, or any other written notes or records to complete the observation tool. In cases when the antenatal care book is used, the enumerator will politely request for the book from the patient. The enumerator will only ask for the book at a time when it is not disruptive to other processes.
    f. If the enumerator is satisfied with the checklist, all information will then be fed into the tablet. Information in the tablet must be identical to that on the paper form.
4) Protocol in case of emergencies:
    a. Protocol for enumerators will be to not intervene in the care provided to the woman, unless in the case of a life-threatening emergency that could benefit directly from the enumerator's involvement. Should the enumerator observe gross mismanagement of a patient and he/she feels that the situation is life threatening, the enumerator may offer advice and/or assistance.
    b. If an enumerator intervenes, they will do so in their capacity as a medical expert, not as a research observer. They must be medically qualified to intervene in the specific situation, that is, have had the necessary training to be qualified to provide care. If

possible, the enumerator should call the study team leader for advice on the way forward before intervening. All instances of enumerators offering assistance to health workers will be well documented. Enumerators should frequently but politely remind the health workers at the facility that they are only observers and not present to provide care or assistance.

Process evaluation: We will use program administrative data to assess the implementation of the intervention until study activities are complete in intervention facilities. This data is routinely collected by Jacaranda Health.

5.8. Does the study involve the use of deception and/or incomplete disclosure?
☒ **No** ☐ **Yes:** *If yes, explain the use of deception/incomplete disclosure and describe why it is necessary to achieve the goals of the study.*

5.9. When all research-related study procedures are complete, are there plans for long-term follow up?
☒ **No** ☐ **Yes:** *If yes, indicate what data will be collected during this period.*

5.10. Does the study involve the collection of specimens (e.g. blood, cells, tissues, fluids, secretions, recombinant or synthetic nucleic acids, biological toxins, bacteria, virus, fungi, etc.)
☒ **No** ☐ **Yes:** *If yes, indicate the COMS Registration Number or plans to obtain COMS approval.*

5.11. Does the study involve the use of existing data, documents, records, and/or specimens for secondary analysis?
☐ **No** ☒ **Yes:** *If yes, indicate how, when, where, and from whom data, documents, records, and/or specimens will be obtained.*

Enumerators will use the Register Data Form to extract data from registers in consultation with facility in-charges and head midwives. These registers are physical books kept at health facilities. The data will cover the entire period from 12-month prior to the baseline up to 12 months after the endline. Facility registers will not leave study facilities. The data extracted from these registers will be shared via Harvard Dropbox with the Harvard study team.

The study team will also use administrative program data collected by Jacaranda Health as part of the process evaluation to assess the implementation of the intervention. The data will be shared by Jacaranda Health with the study team.

5.12. Are there provisions for medical and/or psychological support resources available to participants (e.g., in the event of incidental findings, research-related stress)?
☐ **No** ☒ **Yes:** *If yes, describe the provisions and their availability.*

For participants in the patient surveys, in cases of stillbirth, miscarriage, or infant loss, the study team will inform participants about the option to contact "Still A Mum," a hotline that provides psychological support.

5.13. Describe the data and safety monitoring plan for the study. This plan should outline how study progress will be monitored throughout the lifecycle of the research to ensure the safety of subjects, as well as the integrity and confidentiality of data.

Investigators at Harvard will hold regular management-level meetings/calls with implementation partners in Kenya to discuss unexpected negative outcomes, interim results, protocol modifications, data monitoring, etc. In-person oversight from the management team will be ongoing throughout the study. The Research Manager at IPA will stay in frequent communication with the investigators about any issues related to data quality, adverse events and protocol modifications.

All survey data will be submitted on a daily basis to the SurveyCTO encrypted server and downloaded, backed up, and maintained on a secure, encrypted server managed by IPA. The data will be stored in password protected directories. Data on patients and health workers will be stripped of their identifying information and subjects will be given an alphanumeric code which will be used to identify them. The MS-Excel file with the key to the code of names associated with their respective alphanumeric codes will be kept on an electronic storage medium (ex. flash drive) and will be kept in a locked storage unit. Only the researchers and assistants associated with this study will have access to this document. All electronic data will be stored on an encrypted, secure online server and password protected. Computers holding subject data will also be password protected. A back-up drive containing a copy of all the data will be securely kept in a separate location.

5.14. Are there any anticipated circumstances under which participants will be withdrawn from the research without their consent?

☒ **No** ☐ **Yes:** *If yes, describe the circumstances for withdrawal as well any associated procedures to ensure orderly termination, appropriate referrals, and/or follow-up care.*

6. Recruitment Methods ☐ N/A. *Skip to next section.*

Upload recruitment materials to the "Local-Site Documents" page in ESTR.

6.1. Indicate how, when, where, and by whom participants will be recruited. Provide a list of materials used to recruit participants, e.g., emails, posters, and/or scripts here.

Participants will be recruited as follows. Recruitment scripts are attached to this application.

Health facilities: Prior to beginning work in each county, an approval letter will be signed by the county health director. A research team member will meet with the medical supervisor or maternity in-charge at each facility, present a copy of the letter, and discuss the study. This will only occur in the baseline.

Health workers: At each health facility and prior to conducting consent, nurses and midwives who work in the maternity wards will be recruited into the study by an enumerator for health worker interviews. In the endline, in addition to health worker interviews, other medical providers at maternity ward will be recruited into the study by an enumerator for delivery observation.

Women giving birth: We will recruit women at admission, a time when they are stable during labor, or discharge prior to conducting consent by an enumerator. This will only occur in the endline.

Women receiving antenatal care: At each facility and prior to conducting consent, women receiving antenatal care will be recruited into the study by an enumerator. This will only occur in the baseline.

7. Consent Process

Upload consent form(s) and debriefing materials, if applicable, to the "Local-Site Documents" page in ESTR.

7.1. Describe how the research team will invite participants to take part in the research and obtain consent to participate. If the research team will not obtain informed consent, provide justification for requesting a waiver or alteration of consent (and/or parental permission).

The consent forms and recruitment scripts for medical supervisors, maternity in-charges, health workers, women admitted for labor and delivery, and women receiving antenatal care are attached.

Health facility assessment consent form: The facility leaders of health facilities will be asked to participate in interviews to capture facility-level characteristics in the baseline. Agreement for participation of facilities will also be obtained before data collection activities begin. Agreement will be obtained from the facility medical supervisor for participation of the facility in the assessment. If unavailable, agreement will be obtained from whoever is most knowledgeable about deliveries and has worked at the health center for at least three months.

Pregnant women consent form: Women who are at the health facility for antenatal care will be consented while they are waiting to see the providers or after their visit. An enumerator will approach the woman, describe the study to her, and read the consent form. Women who consent will be asked to sign or put a thumbprint on the consent form, depending on their ability. Women who consent to participate in the study will be provided an information and consent form.

Health worker consent form: Health workers that provide delivery services will be oriented to the study procedures by study enumerators before any observation or interview starts. Health workers will be provided an information and consent form. If a new health worker arrives, they will be oriented to the study and consent will be sought before data collection. Health workers will be free to ask questions about the study or refuse participation and opt-out at any point without any impact on their employment at the facility.

Women admitted for labor and delivery consent form: In the endline, we will consent women from maternity wards at admission or a time when they are stable during labor or after delivery. An

enumerator will approach the woman, describe the study to her, and read the consent form. Women who consent will be asked to sign or put a thumbprint on the consent form, depending on their ability. Women who consent to participate in the study will be provided an information and consent form. We will only ask for consent from women who are stable and able to consent for themselves. In some situations, a woman may be unable to give consent upon arrival to the facility but may later be sufficiently stable to give consent. In this situation, an enumerator will approach the woman once she is stable and able to give consent. Women will not be asked to consent if they are unable to speak or focus on the consent process due to pain or stress from labor.

7.2. For participants under the local age of majority (e.g., 18 years old in Massachusetts), who may reach this milestone during the course of their participation in the study, describe plans to obtain informed consent as parental permission and/or child assent would no longer be valid for such participants. ☐ N/A

Those under 18 years of age (but at least 15 years of age) who are considered a mature minor in Kenya will participate. Mature minors are those who are married, a mother, pregnant, or a household head who are under 18 years of age. Mature minors are allowed to consent on their own without parental permission.

7.3. Describe how the research team will document the consent process (e.g., participant/researcher will both sign and date the consent document; participants will thumbprint the consent document; electronic consent will be obtained and associated with the participant's research record). If the research team will not obtain signature and date, provide justification for requesting a waiver or alteration of documentation of consent (and/or parental permission).

The consent process for the health facility assessment will not involve obtaining signatures from every health worker involved because this is not human subjects research but, rather, is focused on the health facility. We will obtain the signature from one facility representative (e.g., facility in-charge).

The consent process for health workers will not involve obtaining signatures. This way, no personal identifying information for health workers will be saved on paper records (i.e., consent forms) after the data collection period is over. Identifying information collected electronically will only be used to link data collected from different surveys and will not be shared outside of the research team. This will help to avoid a situation where a health worker in the study is blamed for poor quality health care or poor health outcomes observed during direct observation of patient care.

The consent process for antenatal and maternity patients will involve obtaining signatures. It is possible that some of the study participants may not be literate. If the participant is not literate or otherwise unable to consent using a signature, there will be a provision to consent using a thumbprint, rather than signature. In the case that a woman consents using a thumbprint, it will also be required to obtain the signature of a witness who can confirm that the consent has been explained and that her participation is voluntarily. A witness is anyone in the facility who is literate. The enumerator may serve as the witness.

7.4. If the research involves remote or electronic consent, describe how the research team will collect and maintain consent documentation (e.g., all pages of the signed consent form will be returned digitally and associated with the participant's research record; if the requirement for written documentation (signature) is waived, the consent process will be documented in a log; FDA-regulated research will include an identity verification process). ☒ N/A

7.5. Will participants be offered a copy of the consent information?
☒ **Yes** ☐ **No:** *If no, explain why not.*

7.6. If consent will be obtained in a language other than English, identify the language(s) that consent information will be provided, who will be responsible for translation, and the provisions for communicating this information to participants. ☐ N/A

The patient consent forms will be written in English and translated into Swahili. The consent form will be read aloud by the field researcher in the language the patient prefers.

7.7. If the research involves deception and/or incomplete disclosure, describe the debriefing process. Explain when participants will be debriefed, who will debrief them, and how they will be debriefed. ☒ N/A

7.8. If the research involves secondary use of existing data, documents, records, and/or specimens, and the research team will not obtain consent, describe how consent was originally obtained. Additionally, either upload the original consent form to the ESTR record or confirm that the original consent process obtained participants' permission to share or use their data/specimens for future research projects. ☐ N/A

We will collect data from existing medical records kept at health facilities (e.g., the MOH333 Maternity Register). Enumerators will enter the register data into tablets without any personally identifying information (i.e., name, date of birth, or contact information) and we will not obtain consent. In the endline, for the patients who consent to being observed, enumerators will also obtain patients' consent to access their medical records in health facilities. For this subset of patients, we will collect data from their health records with identifying information.

We will extract administrative data that Jacaranda Health routinely collects on the implementation of the interventions. We will only extract individually identifiable data from program records for participants that have consented to the study. This will allow us to link data on intervention use with the survey data we collect. For program records without individual consent, we will only collect aggregated data.

8. HIPAA Privacy Protections ☒ N/A. *Skip to next section.*

HIPAA applies to US-based research involving the collection or use of protected health information (PHI) from a hospital, health center (including the Harvard Dental Center), health plan, or health insurance plan (i.e. a covered entity). The Privacy Rule will not directly regulate researchers who are engaged in research within organizations that are not covered entities even though they may gather, generate, access, and share personal health information (PHI). The Privacy Rule applies only to individually identifiable health information held or maintained by a covered entity. Individually identifiable health information that is held by anyone other than a covered entity, including an independent researcher who is not a covered entity, is not protected by the Privacy Rule and may be used or disclosed without regard to the Privacy Rule.

8.1. Explain how the Privacy Rule applies to this specific research project.

8.2. Describe the covered entity involved in this research that holds or maintains the PHI that will be used by the researchers.

8.3. Describe plans for obtaining authorization to access protected health information or provide the rationale for a waiver of authorization.

9. Research Subject to the European Union (EU) General Data Protection Regulation (GDPR)

☒ **N/A.** *Skip to next section.*

GDPR applies to research involving the collection of "personal data" from research subjects who are located in the EU/EEA/UK. This includes biospecimens. The EU/EEA includes the 27 states of the European Union (Austria, Belgium, Bulgaria, Croatia, Republic of Cyprus, Czech Republic, Denmark, Estonia, Finland, France, Germany, Greece, Hungary, Ireland, Italy, Latvia, Lithuania, Luxembourg, Malta, Netherlands, Poland, Portugal, Romania, Slovakia, Slovenia, Spain, & Sweden) and five additional countries: Iceland, Liechtenstein, Norway, Switzerland, & United Kingdom.

9.1. Describe plans to collect and/or obtain "pseudonymized data" (e.g., coded data) and/or identifiable data and/or biospecimens from participants in the EEA/UK.

9.2. Describe plans to destroy "pseudonymized data" (e.g., coded data) and/or identifiable data and/or biospecimens at the conclusion of the study.

9.3. Will data collected from individuals located in the EEA/UK include any of the following? (check all that apply)

☐ Health Information	☐ Trade Union Membership
☐ Racial or Ethnic Origin	☐ Sexual Orientation/Sex Life
☐ Political Opinions	☐ Biometric Data
☐ Religious or Philosophical Beliefs	☐ Genetic Data
☐ Criminal Activity	☐ None of the above

10. Research Subject to the Family Educational Rights and Privacy Act (FERPA)

☒ **N/A.** *Skip to next section.*

FERPA applies to research involving the collection of individually identifiable information from student records or personal education information from an education program (defined as: any program principally engaged in the provision of education, including, but not limited to, early childhood education, elementary and secondary education, postsecondary education, special education, job training, career and technical education, and adult education).

10.1. Describe plans to collect and/or obtain individually identifiable information from student records or personal education information from an education program.

11. Vulnerable Populations ☐ N/A. *Skip to next section.*

11.1. Identify all vulnerable populations (e.g., children; pregnant women, human fetuses, neonates; prisoners; elderly; economically disadvantaged; employees or students of the investigator or sponsor; undocumented individuals; refugees; racial and/or ethnic minorities; illiterate or low-literacy; military personnel; terminally ill; cognitively impaired or mentally ill; persons with a stigmatizing disease or condition, e.g. AIDS/HIV, etc.) and describe safeguards to protect their rights and welfare.

Pregnant women: Women will be read the information and consent form upon arrival to the health facility, with an emphasis on voluntary participation in the study.

Illiterate or low-literacy persons: A provision in the consent form allows for a thumbprint rather than a signature to confirm consent. In the case that a woman consents using a thumbprint, it will also be required to obtain the signature of a witness who can confirm that the consent has been explained and that her participation is voluntarily. A witness is anyone in the facility who is literate.

Persons with stigmatizing disease or condition, e.g., AIDS/HIV: We will collect data on patients' disease status to assess whether certain service procedures are provided as part of quality-of-care indicators. Patient information will be kept confidential following the data collection and storage protocols. In general, there is no differential risk of disclosure by disease status as research protocols will be the same.

12. Risks

Risks may be physical, psychological, social, legal, reputational, and/or financial.

12.1. Describe the reasonably foreseeable risks, discomforts, and/or inconveniences to participants and/or the group/community to which they may belong. Indicate the probability, magnitude, and duration of each risk.

The proposed study procedures pose minimal risk to study participants. Presence of a data collector in the antenatal care clinic or maternity ward may cause slight discomfort to patients and health workers, but all efforts will be taken to minimize any disruption or discomfort caused by the presence of data

collectors. In addition, as with any research study, there is a possibility of a risk of breach of confidentiality despite stringent efforts to keep all data secure.

Patients might be exposed to increased risk of Covid-19 infection by interacting with enumerators at health facilities during the pandemic. To minimize patients' risks, we will provide a face mask to any patient that does not have a mask. Meanwhile, enumerators will wear personal protective equipment when they interact with patients or health workers. Enumerators will recruit participants and conduct interview outdoors as much as possible.

Patients who experience health complications or loss (e.g., in the case of neonatal death) might go through emotional distress and find it difficult to discuss their experience during a survey. During the follow-up phone surveys, we will let participants know that they do not need to continue the survey if talking about their experience is too stressful or traumatic to them. We will also offer such participants information on mental health support services and encourage them to seek care. In cases of stillbirth, miscarriage, or infant loss, we will provide women with information about "Still A Mum", a hotline that provides free psychological support in Kenya.

12.2. Identify whether any of the information collected, if disclosed outside of the research, could reasonably place the participant at risk of criminal or civil liability or be damaging to the participant's financial standing, employability, insurability, or reputation.

We will collect data on health workers' assessment of quality of care and facility readiness. We will also assess health workers' professional knowledge and collect data on quality of maternity care via delivery observations. Disclosure of health workers' identity outside of the research could be damaging to health workers' job security and reputation at work.

We will collect data on patients' disease status, including stigmatizing conditions such as HIV. Disclosure of such information outside of the research could present damage to participants' employability or reputation.

12.3. Outline provisions in place to minimize each risk identified above.

To minimize risk of study data disclosure, enumerators will be trained on study procedures and research ethics. Enumerators will obtain informed consent from health workers or women prior to starting any data collection. They will emphasize that participation is voluntary and that participants may withdraw from the research at any time. Enumerators will interview participants at a private space to protect participants' privacy. No individual facilities, health workers, or women will be identified in any written reports of the findings. If data were shared outside of the certified research team, all personally identifiable information will be removed and responses will be anonymous and only aggregate information will be presented.

For health workers, we will not collect personally identifiable information in the health worker survey or consent form. However, we will collect health worker names to ensure accurate recording of health worker roles in care. The names and corresponding ID numbers of health workers will be recorded on a Health Worker Key document and will be kept securely by IPA Field Manager and solely used for logistical and organizational purposes. This key will be destroyed at the end of study. If data is shared outside of the certified research team, all personal identifying information will be removed and responses will be anonymous and only aggregate information will be presented.

13. Benefits

13.1. Describe the potential benefits to individual participants, if any, and/or society. If there are no direct benefits, state that here. Note: payment/compensation is not a benefit.

While there are no immediate benefits of the study to participants, there are potential benefits of the study to society: results from this study will generate important information on factors that influence maternity care quality and patient health and inform Jacaranda Health's operations as well as other similar programs.

14. Participant Privacy

14.1. Describe provisions to protect participants' privacy (their ability to control and limit the extent, timing, and circumstances of sharing information about themselves with others, e.g., the use of a private interview room) and to minimize any sense of intrusiveness that may be caused by study questions or procedures.

Pregnant women present for antenatal visits & patients admitted for labor and delivery
Enumerators will be instructed to interview patients at a private space to protect participants' privacy. Only the study register and follow-up database will contain identifying information such as name and contact details. This information will enable us to make follow-up calls. Women will be informed that they could skip any question and are free to withdraw consent at any point during the study period. Any personally identifying data will be stored in an encrypted database.

Health workers
No personally identifiable information will be collected in the questionnaires or consent forms. However, a Health Worker Key document with respondent names and corresponding IDs will be used during data collection in order to ensure accurate recording of health worker roles in care. This key will be destroyed at the end of data collection. If data is shared outside of the certified research team, all personally identifiable information will be removed and responses will be anonymous and only aggregate information will be presented.

During the health worker interview, participants will be told that they can skip any question that they find intrusive. All interviews will be conducted individually, in a location that is private.

15. Data Confidentiality

15.1. Indicate the identifiability of the data/specimens: (check all that apply)

☐	Data/specimens will not contain any direct or indirect identifiers (anonymous data), e.g., underlying data did not collect identifiers and/or the data provider will remove the identifiers before sharing with Harvard agents.
☒	Data/specimens will contain direct or indirect identifiers, but the research team will remove them upon receipt (de-identified data).
☒	Data/specimens will contain indirect identifiers (i.e., number, letter, symbol, or combination thereof) and the research team will maintain a key that links identifiers to individual participants (coded data).
☒	Data/specimens will contain direct identifiers (identifiable data).
☐	None of the above; describe:

15.2. Have any identifiable data/specimens been de-identified for use in this research study?
☐ **No** ☒ **Yes:** *If yes, describe how you will prevent any re-identification.*

We will extract data from facility registers without any identifiable information.

For patients who consent to having their labor and delivery observed at health facilities, we will obtain their consent to collect identifiable data from their registers and other facility records so that we could link these patient records with data collected from observations.

15.3. Identify where data/specimens will be stored (e.g., on campus at Harvard or remotely, in a specimen laboratory) and describe the provisions to maintain confidentiality (e.g., password protection, encryption, locked filing cabinets, etc.). Refer to the Investigator Manual and the Harvard Research Data Security Policy for additional information.

Data collected on paper will be immediately converted to electronic data files by the enumerators in the field. The Filed Manager will scan all the paper survey and save to a password-protected app. The files will then be transferred to a larger locked storage closet specific to this research study at the locked IPA Kenya office, where they will be held until the completion of all research-associated publications.

Data collected on tablets will be uploaded to a secure, encrypted online server that is password protected. The tablets and computers holding subject data will also be password protected. All survey data will be backed up and maintained on a secure, encrypted server managed by IPA.

Data on patients will be stripped of their identifying information and subjects will be given an alphanumeric code which will be used to identify them. The MS-Excel file with the key to the code of names associated with their respective alphanumeric codes will be kept in electronic storage maintained by IPA.

For health workers, we will not collect personally identifiable information in the health worker survey. Instead, we will use a Health Worker ID-to-Name Key document with respondent names and corresponding IDs to link health worker data collected from different surveys. The file with the ID-to-Name Key data will be kept on a secure, encrypted server managed by IPA and kept separate from the research data. The codes will be used both on the tablets and in the Labor & Delivery Observation checklist (paper-based data collection tool). The Name-to-Key Data will only be used by IPA and will not be shared with the Harvard team.

The Harvard team will receive identifiable data. All data will be uploaded to Harvard Dropbox by local collaborators and will be accessible to research team members via a Dropbox account with a strong secure password.

15.4. Indicate whether any data/specimens will be transferred/transmitted and describe the plan to share the data/specimens (e.g., outside of Harvard, to other researchers, to collaborators). Indicate who may request access and how. If data/specimens will be transferred/transmitted/shared, describe how, when, and to whom.

IPA, our implementation partner, commits to comply with the principles of data protection set forth in the Kenya Data Protection Act, 2019. IPA may transfer personal data inside Kenya or to the United States for the purposes of this research project only. IPA has in place security measures, such as encrypted software, and limits access to personal data on a need-to-know basis.

Data will only be collected by the field enumeration team. The Harvard team will use Harvard Dropbox for data storage. No files with identifiable information will be transmitted by email. Files will be uploaded to Harvard Dropbox and will be accessible to research team members via a Dropbox account with a strong secure password.

Tablet computers will be used to collect data for health worker interviews and facility level interviews. These will have an up-to-date operating system, be encrypted with a strong password, and include antivirus software. After all data collection is completed, tablet computers will be reset to factory defaults to securely remove all data.

15.5. Indicate whether participants' permission will be obtained to share their data/specimens and/or use their data/specimens in other future research projects.

We have no plans to use information in future research projects so no permission will be sought.

15.6. Will recordings be obtained in this research study?

☒ **No** ☐ **Yes:** *If yes, select the kind(s) of recording and describe the transcription and destruction plan.*

☐ Audio recording	☐ Video recording
Transcription plan: Interviews may be recorded for quality control purposes by IPA. Respondents are free to refuse audio recording with no consequences. These recordings will not be transcribed or shared with the research team.<br>Destruction plan: Audio recordings will be destroyed after completion of audio audits.	

15.7. Indicate who is responsible for data/specimen management and how the research team and/or other collaborators are permitted access to information.

IPA is responsible to put in place the data protection systems outlined, then will share permissions with the team of primary researchers who will jointly manage the data on Harvard Dropbox. To access the information, the researchers must log into Harvard Dropbox on their computer or online. Any collaborators would also have to log into the cloud to access the dataset.

15.8. Indicate how long data/specimens will be stored and describe the plans at the end of the storage period (e.g., are data/specimens destroyed, returned to data/specimen provider, etc.).

All books, records and reports will be kept for five years following the end of the study period.

16. Data/Statistical Analyses Plan

16.1. Describe plans for analysis (including the statistical method, if applicable).

Statistical analysis will consist of summarizing data with descriptive statistics and t-tests, and evaluating the effectiveness of the intervention using multiple regression.

16.2. Is there a sample size/power calculation?

☐ **No** ☒ **Yes:** *If yes, describe the calculation and the scientific rationale, and, if applicable, by site and key characteristics such as participant demographics*.

The baseline sample size is based on the power calculation for postnatal care within 6 weeks of delivery, which is our primary outcome for patient care seeking. Based on Jacaranda’s internal data, we assume that 61% of women will receive any postnatal care visit within 6-8 weeks of delivery in the absence of the intervention. Based on health information system volume data, we would expect to see 120 ANC patients new to the intervention in study facilities in a given month and we expect at least 80% of them will be enrolled into the PROMPTS intervention. We plan to enroll women over at least a one-month period to reach this target. Assuming that we can track 75% of these women postpartum regarding post-delivery outcomes, we would expect to follow roughly 58 women for this outcome in each facility. Assuming an ICC of 0.01, the minimum detectable impact is an increase in postnatal care receipt of 7 percentage points or a 11% increase in postnatal care seeking.

The endline sample size is based on the power calculation for quality of care during labor, delivery and immediate postpartum, which is our primary health worker outcome. We assume that we will be able to observe at least 100 complete deliveries during six weeks of observation in each facility at the endline. Based on the PIs recent work in Uganda and Kenya, which used a very similar technique of clinical observations of deliveries in similar health facilities, we assume that average quality of care for the control group will be 0.58 (i.e., health workers will perform just over half of the important aspects of quality of care) with a standard deviation of 0.13. Assuming an intra-cluster correlation (ICC) of 0.35 (based on baseline data from our previous work), a cluster size of 100 patients, 20 clusters per arm, alpha of 0.05, and power of 0.80, the minimum detectable impact would be 6.88 percentage point increase in quality of care or an 11.9% increase in the quality-of-care index.

17. Costs and Compensation ☐ **N/A.** *Skip to next section.*

17.1. Identify any costs that participants may incur during the study, including transportation costs, childcare, or other out-of-pocket expenses.

There are no other anticipated costs that participants may incur during the study.

17.2. Identify remuneration that participants may receive during the study. Specify the amount, timing of disbursement, and method (e.g. money, gift cards, in-kind, incentives, raffles, and transportation). Describe how compensation will be calculated and paid if a participant withdraws. If any participant will receive a single payment more than $100, or $600 or more in one calendar year, refer to Harvard University Financial Policy on Human Subject Payments.

Facilities will be given a small gift (such as a clock or infrared thermometers) for participation in the study. All participating facilities will receive the same gift with the value around KES 3500.

Health workers will be offered a small token of appreciation in the form of airtime with the value of around KES 200 for participating in the health worker surveys.

Women recruited from the ANC clinic will receive a small token of appreciation in the form of airtime with the value of KES 100 per survey (baseline survey and two follow-up calls).

18. Sharing Study Results ☒ **N/A.** *Skip to next section.*

18.1. Describe the plan to share study results with individual participants, the participant group/community, and/or others.

We plan to share a report of the findings with directors of health, department of family health, leadership of other counties, and facilities through technical working groups.

19. Research Related Injuries ☐ **N/A.** *Skip to next section.*

19.1. Describe plans for medical care and compensation for research-related injuries.

Enumerators might be exposed to increased risk of Covid-19 infection by working in health facilities. To minimize these risks, we will provide personal protective equipment to all study enumerators. We will encourage enumerators to recruit participants and conduct interviews outdoors as much as possible. We will provide enumerators with additional face masks to be distributed to any patient or health worker they interview that does not have a mask.

20. Reportable Events

20.1. Outline plans for communicating reportable events to the IRB, Sponsor, or others as applicable (e.g., adverse events, unanticipated problems involving risks to participants or others, breach of confidentiality).

If a reportable event directly related to the research activities occurs, the enumerators at the facility will immediately notify the field manager who will escalate the information to the Principal Investigator at the Harvard Chan School. The reportable event will be reported to both local and Harvard IRBs by the investigators within five business days.

21. Regulatory Compliance

21.1. Describe plans for monitoring regulatory compliance. The monitoring plan should include how you will ensure proper record keeping, retention of required regulatory documents and participant files, and adherence to the IRB-approved protocol and/or IRB policies and procedures. Monitoring plans should describe 1) who is responsible for file maintenance, 2) what will be maintained, 3) how often files will be reviewed and using what method, and 4) where documentation will be retained (for both Regulatory Documents and Participant files). The IRB recommends the use of QIP's Study Management Tools to monitor regulatory compliance and organize study documents.

Compliance to the IRB policies and procedures will be a priority during field data collection and afterwards and will include the following:

I. All researchers will have completed the NIH Human Subjects training, CITI or equivalent
II. Anyone outside of this research team must only view the de-identified dataset after the personal identification information has been removed
III. Field management staff and the enumeration team will be trained in the importance of maintaining protocols for consent and data collection
IV. Copies of consent forms will be kept by IPA and field management staff will ensure that these have been collected from each respondent

22. Data or Biospecimen Sharing ☒ **N/A.** *Skip to next section.*

If you plan to establish a repository, please submit a separate application using the HLC Repository Protocol Template.

22.1. Describe the plan to send data/specimens to research collaborators outside of Harvard.

☐ **N/A**

22.2. Describe the plan to receive data/specimens <u>from</u> collaborators outside of Harvard.
☐ **N/A**

23. Clinical Trials ☒ **N/A.** *Skip to next section.*
Complete this section for clinical trials, including NIH funded clinical trials or applicable clinical trials (ACT) under the FDA Amendments Act. To determine if a study meets the definition of a clinical trial, follow the guidance in the "Preparing the Research Protocol" section of the Investigator Manual.

23.1. Describe how this study meets the definition of a clinical trial.

23.2. Describe plans for registering this project in a clinical trials registry, e.g., clinicaltrials.gov. If available, provide the registry record number.

23.3. Describe plans for posting the clinical trial consent form on a publicly available federal website per federal requirements in the Common Rule (45§46.116(h)).

24. Device *This section <u>should</u> be completed if the study involves the use of any device on/in/with human subjects, and/or the use any device utilizing human specimens, which meets the FDA definition of a medical device.* ☒ **N/A.** *Skip to next section.*

24.1. Describe the device, including the generic or common name, brand name (if applicable), purpose, function/operation, and whether it is an implant. Indicate who is providing this device for research use.

24.2. Indicate the FDA status of the device as it is being used for the proposed research:
☐ FDA-approved device being used "on-label" (i.e., FDA-approved purpose, population, manner).
☐ FDA-approved device that is being used "off-label" (i.e., for a different purpose, population, or in a different manner than approved).
☐ Not approved by the FDA.

24.3. Indicate the IDE Status of this device:
☐ The use of this device has an IDE.
☐ The use of the device qualifies for an Abbreviated IDE.
☐ The use of the device is exempt from the IDE requirements.

24.4. Has the FDA made a determination as to whether the device is Significant Risk or Non-Significant Risk? ☐ **No** ☐ **Yes:** *If yes, indicate the FDA's determination.*

24.5. Describe plans for storage control, and dispending of the product so that (1) only authorized investigators will use the product; (2) the product will only be used in participants who have provided consent, and (3) there will be documented tracking of each product, including unique identifiers and any return/disposal.

25. Drug/Biologic *This section* *should* *be completed if the study involves the use of any drug/biologic on/in/with human subjects which meets* *the FDA definition of a drug/biologic*. ☒ **N/A.** *Skip this section.*

25.1. Describe the drug or biologic, including the generic or common name, brand name (if applicable), dosing, route of administration, number of doses, timing of administration. Indicate who is providing the drug, biologic, supplement for research use.

25.2. Indicate the IND Status of this drug or biologic and who holds the IND: (select one)

☐	There is an IND approval from the FDA for the use of this item.
	The IND is held by:
	The IND number is:
☐	An IND application has been, or will be, submitted to the FDA.
	The IND will be held by:
	The IND number is:
☐	An IND approval is not required.

25.3. Describe how dispensing, delivery and administration will be performed, and by whom. Include information about control (e.g., locked storage), tracking (e.g., lot number, returned pills), documentation, storage, and return/disposal.

1. World Health Organization. *Trends in Maternal Mortality 2000 to 2017: Estimates by WHO, UNICEF, UNFPA, World Bank Group and the United Nations Population Division*.; 2019. https://apps.who.int/iris/handle/10665/327596
2. UN Interagency Group for Child Mortality Estimation. Mortality rate, neonatal (per 1,000 live births). Published 2019. Accessed February 16, 2021. https://data.worldbank.org/indicator/SH.DYN.NMRT
3. Kruk ME, Gage AD, Joseph NT, Danaei G, García-Saisó S, Salomon JA. Mortality due to low-quality health systems in the universal health coverage era: a systematic analysis of amenable deaths in 137 countries. *The Lancet*. 2018;392(10160):2203-2212. doi:10.1016/S0140-6736(18)31668-4
4. Munabi-Babigumira S, Glenton C, Lewin S, Fretheim A, Nabudere H. Factors that influence the provision of intrapartum and postnatal care by skilled birth attendants in low- and middle-income countries: A qualitative evidence synthesis. *Cochrane Database of Systematic Reviews*. 2017;2017(11). doi:10.1002/14651858.CD011558.pub2
5. Ameh CA, Mdegela M, White S, Van Den Broek N. The effectiveness of training in emergency obstetric care: A systematic literature review. *Health Policy and Planning*. 2019;34(4):257-270. doi:10.1093/heapol/czz028
6. Rowe AK, de Savigny D, Lanata CF, Victora CG. How can we achieve and maintain high-quality performance of health workers in low-resource settings. *Lancet*. 2005;366:1026-1035. doi:10.1016/S0140-6736(05)
7. Feyissa GT, Balabanova D, Woldie M. How effective are mentoring programs for improving health worker competence and institutional performance in africa? A systematic review of quantitative evidence. *Journal of Multidisciplinary Healthcare*. 2019;12:989-1005. doi:10.2147/JMDH.S228951
8. Schwerdtle P, Morphet J, Hall H. A scoping review of mentorship of health personnel to improve the quality of health care in low and middle-income countries. *Globalization and Health*. 2017;13(1). doi:10.1186/s12992-017-0301-1
9. Vasan A, Mabey DC, Chaudhri S, Epstein HAB, Lawn SD. Support and performance improvement for primary health care workers in low- and middleincome countries: A scoping review of intervention design and methods. *Health Policy and Planning*. 2017;32(3):437-452. doi:10.1093/heapol/czw144
10. Thaddeus S, Maine D. Too far to walk: Maternal mortality in context. *Social Science & Medicine*. 1994;38(8):1091-1110. doi:10.1016/0277-9536(94)90226-7
11. Actis Danna V, Bedwell C, Wakasiaka S, Lavender T. Utility of the three-delays model and its potential for supporting a solution-based approach to accessing intrapartum care in low- and middle-income countries. A qualitative evidence synthesis. *Global Health Action*. 2020;13(1):1819052. doi:10.1080/16549716.2020.1819052
12. Soubeiga D, Gauvin L, Hatem MA, Johri M. Birth Preparedness and Complication Readiness (BPCR) interventions to reduce maternal and neonatal mortality in developing countries: Systematic review and meta-analysis. *BMC Pregnancy and Childbirth*. 2014;14(1):1-11. doi:10.1186/1471-2393-14-129
13. Kenya National Bureau of Statistics, Ministry of Health/Kenya, National AIDS Control Council/Kenya, Kenya Medical Research Institute, National Council for Population and Development/Kenya. *Kenya Demographic and Health Survey 2014*.; 2015.
14. Sondaal SFV, Browne JL, Amoakoh-Coleman M, et al. Assessing the Effect of mHealth

Interventions in Improving Maternal and Neonatal Care in Low- and Middle-Income Countries: A Systematic Review. Li D, ed. *PLOS ONE*. 2016;11(5):e0154664. doi:10.1371/journal.pone.0154664
15. Feroz A, Perveen S, Aftab W. Role of mHealth applications for improving antenatal and postnatal care in low and middle income countries: a systematic review. *BMC Health Services Research*. 2017;17(1):704. doi:10.1186/s12913-017-2664-7
16. Lee SH, Nurmatov UB, Nwaru BI, Mukherjee M, Grant L, Pagliari C. Effectiveness of mHealth interventions for maternal, newborn and child health in low– and middle–income countries: Systematic review and meta–analysis. *Journal of Global Health*. 2016;6(1). doi:10.7189/jogh.06.010401
