# Supplementary material for: Impact evaluation of a digital health platform empowering Kenyan women across the pregnancy-postpartum care continuum: A cluster randomized controlled trial
Source: PLoS Med. 2025 Feb 3;22(2):e1004527. doi: 10.1371/journal.pmed.1004527 (PMC11835334; doi:10.1371/journal.pmed.1004527)
Supplement: S1 Checklist — (PDF) [file pmed.1004527.s002.pdf]

S1 Checklist. Consolidated Standards of Reporting Trials (CONSORT) Checklist of Information to Include When Reporting a Randomized Trial, with Extensions for Cluster-Randomized Trials

Section/Topic	Item No	Standard Checklist item	Extension for Cluster Designs	Reported in…
**Title and abstract**				
	1a	Identification as a randomized trial in the title	Identification as a cluster randomized trial in the title	Title Page
	1b	Structured summary of trial design, methods, results, and conclusions (for specific guidance see CONSORT for abstracts)		Abstract; see S2 Checklist for further details
**Introduction**				
Background and objectives	2a	Scientific background and explanation of rationale	Rationale for using a cluster design	Introduction
	2b	Specific objectives or hypotheses	Whether objectives pertain to the cluster level, the individual participant level, or both	Introduction
**Methods**				
Trial design	3a	Description of trial design (such as parallel, factorial) including allocation ratio	Definition of cluster and description of how the design features apply to the clusters	Methods > Study Design, Intervention, and Participants
	3b	Important changes to methods after trial commencement (such as eligibility criteria), with reasons		N/A
Participants	4a	Eligibility criteria for participants	Eligibility criteria for clusters	Methods > Study Design, Intervention, and Participants > Health Facility Eligibility; Methods > Study Design, Intervention, and Participants > Participant Recruitment and Eligibility
	4b	Settings and locations where the data were collected		Methods > Study Design, Intervention, and Participants > Recruitment and Eligibility;

				Methods > Procedures Data Collection; Methods > Procedures > Enrollment in PROMPTS; Fig 2
Interventions	5	The interventions for each group with sufficient details to allow replication, including how and when they were actually administered	Whether interventions pertain to the cluster level, the individual participant level, or both	Methods > Study Design, Intervention, and Participants > Intervention
Outcomes	6a	Completely defined pre-specified primary and secondary outcome measures, including how and when they were assessed	Whether outcome measures pertain to the cluster level, the individual participant level, or both	Methods > Outcomes
	6b	Any changes to trial outcomes after the trial commenced, with reasons		Methods > Outcomes
Sample size	7a	How sample size was determined	Method of calculation, number of clusters(s) (and whether equal or unequal cluster sizes are assumed), cluster size, a coefficient of intracluster correlation (ICC or k), and an indication of its uncertainty	Methods > Statistical Analysis > Sample Size and Power
	7b	When applicable, explanation of any interim analyses and stopping guidelines		N/A
Randomization				
Sequence generation	8a	Method used to generate the random allocation sequence		Methods > Randomization and Masking; S1 Text
	8b	Type of randomization; details of any restriction (such as blocking and block size)	Details of stratification or matching if used	Methods > Randomization and Masking; S1 Text
Allocation concealment mechanism	9	Mechanism used to implement the random allocation sequence (such as sequentially numbered containers), describing any steps taken to conceal the	Specification that allocation was based on clusters rather than individuals and whether allocation concealment (if any) was at the cluster	Methods > Randomization and Masking

		sequence until interventions were assigned	level, the individual participant level, or both	
Implementation	10	Who generated the random allocation sequence, who enrolled participants, and who assigned participants to interventions	Replaced by 10a, 10b, and 10c	
	10a		Who generated the random allocation sequence, who enrolled clusters, and who assigned clusters to interventions	Methods > Randomization and Masking
	10b		Mechanism by which individual participants were included in clusters for the purposes of the trial (such as complete enumeration, random sampling)	Methods > Study Design, Intervention, and Participants > Participant Recruitment and Eligibility
	10c		From whom consent was sought (representatives of the cluster, or individual cluster members, or both) and whether consent was sought before or after randomization	Methods > Procedures > Data Collection; Methods > Procedures > Enrollment in PROMPTS; Methods > Ethics and Safety Statement
Blinding	11a	If done, who was blinded after assignment to interventions (for example, participants, care providers, those assessing outcomes) and how		N/A
	11b	If relevant, description of the similarity of interventions		N/A
Statistical methods	12a	Statistical methods used to compare groups for primary and secondary outcomes	How clustering was taken into account	Methods > Statistical Analysis
	12b	Methods for additional analyses, such as subgroup analyses and adjusted analyses		Methods > Statistical Analysis
**Results**				

Participant flow (a diagram is strongly recommended)	13a	For each group, the numbers of participants who were randomly assigned, received intended treatment, and were analyzed for the primary outcome	For each group, the numbers of clusters that were randomly assigned, received intended treatment, and were analyzed for the primary outcome	Results > Sample Attrition and Characteristics and Fig 1
	13b	For each group, losses and exclusions after randomization, together with reasons	For each group, losses and exclusions for both clusters and individual cluster members	Results > Sample Attrition and Characteristics; S3 Table
Recruitment	14a	Dates defining the periods of recruitment and follow-up		Methods > Procedures > Data Collection; Fig 1
	14b	Why the trial ended or was stopped		Methods > Procedures > Data Collection
Baseline data	15	A table showing baseline demographic and clinical characteristics for each group	Baseline characteristics for the individual and cluster levels as applicable for each group	Results > Sample Attrition and Characteristics; Table 2, S4 Table, S5 Table
Numbers analyzed	16	For each group, number of participants (denominator) included in each analysis and whether the analysis was by original assigned groups	For each group, number of clusters included in each analysis	Methods > Statistical Analysis; Results > Sample Attrition and Characteristics; S4 Table, S5 Table
Outcomes and estimation	17a	For each primary and secondary outcome, results for each group, and the estimated effect size and its precision (such as 95% confidence interval)	Results at the individual or cluster level as applicable and a coefficient of intracluster correlation (ICC or k) for each primary outcome	Results > Intervention Impact; Tables 4 and 5; Figs 4 and 5; ICC not reported
	17b	For binary outcomes, presentation of both absolute and relative effect sizes is recommended		Results > Intervention Impact and Discussion > Implications and Future Directions
Ancillary analyses	18	Results of any other analyses performed, including subgroup		Results > Intervention

		analyses and adjusted analyses, distinguishing pre-specified from exploratory		Impact; Tables 4 and 5; Fig 5
Harms	19	All important harms or unintended effects in each group (for specific guidance see CONSORT for harms)		N/A
**Discussion**				
Limitations	20	Trial limitations, addressing sources of potential bias, imprecision, and, if relevant, multiplicity of analyses		Discussion > Strengths and Limitations
Generalizability	21	Generalizability (external validity, applicability) of the trial findings	Generalizability to clusters and/or individual participants (as relevant)	Discussion > Implications and Future Directions
Interpretation	22	Interpretation consistent with results, balancing benefits and harms, and considering other relevant evidence		Discussion
**Other information**				
Registration	23	Registration number and name of trial registry		Abstract > Trial Registration and Methods
Protocol	24	Where the full trial protocol can be accessed, if available		S1 Protocol
Funding	25	Sources of funding and other support (such as supply of drugs), role of funders		Disclosed to Journal
