# Supplementary material for: Impact evaluation of a digital health platform empowering Kenyan women across the pregnancy-postpartum care continuum: A cluster randomized controlled trial
Source: PLoS Med. 2025 Feb 3;22(2):e1004527. doi: 10.1371/journal.pmed.1004527 (PMC11835334; doi:10.1371/journal.pmed.1004527)
Supplement: S2 Checklist — (PDF) [file pmed.1004527.s003.pdf]

S2 Checklist. CONSORT Checklist for Abstracts to Reports of Randomized Trials, with Extensions for Cluster-Randomized Trials

Section/Topic	Standard Checklist Item	Extension for Cluster Designs	Reported in…
Title	Identification of the study as randomized	Identification of study as cluster randomized	Title page
Trial design	Description of the trial design (for example, parallel, cluster, non-inferiority)		Abstract > Methods and Findings
Methods:			
Participants	Eligibility criteria for participants and the settings where the data were collected	Eligibility criteria for clusters	Insufficient room in abstract; reported in main text in Methods > Study Design, Intervention, and Participants > Health Facility Eligibility; Methods > Study Design, Intervention, and Participants > Participant Recruitment and Eligibility
Interventions	Interventions intended for each group		Abstract > Methods and Findings
Objective	Specific objective or hypothesis	Whether objective or hypothesis pertains to the cluster level, the individual participant level, or both	Abstract > Methods and Findings
Outcome	Clearly defined primary outcome for this report	Whether the primary outcome pertains to the cluster level, the individual participant level or both	Abstract > Methods and Findings
Randomization	How participants were allocated to interventions	How clusters were allocated to interventions	Insufficient room in abstract; reported in main text in Methods > Randomization and Masking
Blinding (masking)	Whether or not participants, care givers, and those assessing the		Abstract > Methods and Findings

	outcomes were blinded to group assignment		
Results:			
Numbers randomized	Number of participants randomized to each group	Number of clusters randomized to each group	Number of clusters randomized to each group implied in Abstract > Methods and Findings
Numbers analyzed	Number of participants analyzed in each group	Number of clusters analyzed in each group	Insufficient room in abstract to disaggregate by group; reported in main text in Results > Sample Attrition and Characteristics
Outcome	For the primary outcome, a result for each group and the estimated effect size and its precision	Results at the cluster or individual level as applicable for each primary outcome	Abstract > Methods and Findings
Harms	Important adverse events or side effects		N/A
Conclusions	General interpretation of the results		Abstract > Conclusions
Trial registration	Registration number and name of trial register		Abstract > Trial Registration
Funding	Source of funding		Disclosed to Journal
