# Supplementary material for: Impact evaluation of a digital health platform empowering Kenyan women across the pregnancy-postpartum care continuum: A cluster randomized controlled trial
Source: PLoS Med. 2025 Feb 3;22(2):e1004527. doi: 10.1371/journal.pmed.1004527 (PMC11835334; doi:10.1371/journal.pmed.1004527)
Supplement: S1 Text — (PDF) [file pmed.1004527.s004.pdf]

S1 Text. Technical Overview of Facility-Level Randomization

The randomization algorithm was implemented by the research team at Harvard. Using the randomize command in Stata, we iterated through 1:1 treatment-to-control randomization assignments until the corresponding balance p-value exceeded 0.7, with a maximum of 2,000 iterations permitted. The balance p-value accounted for facility-level caesarean birth frequency (i.e., proportion of total births conducted by caesarean section) and perinatal mortality (i.e., proportion of total stillbirths and live births ending in stillbirth or neonatal death within one week).

After running the randomization algorithm, facility locations were visualized on the map and reviewed with the team at Jacaranda Health. Though the randomization procedure was not constrained by geographic location, facilities assigned to different study arms were found to have a minimum distance of 10 kilometers between them. The distance between each facility and the nearest facility of the opposite arm was, on average, 32 kilometers. Even for facilities proximate to one another, transit times often could still be substantial.
