# Supplementary material for: Impact evaluation of a digital health platform empowering Kenyan women across the pregnancy-postpartum care continuum: A cluster randomized controlled trial
Source: PLoS Med. 2025 Feb 3;22(2):e1004527. doi: 10.1371/journal.pmed.1004527 (PMC11835334; doi:10.1371/journal.pmed.1004527)
Supplement: S2 Text — (PDF) [file pmed.1004527.s005.pdf]

S2 Text. Pre-Registered Outcomes Excluded from the Main Manuscript

The following two pre-registered primary outcomes and five pre-registered secondary outcomes were excluded from the main manuscript for the reasons articulated below.

Primary Outcomes

- Share of antenatal, postpartum, and neonatal danger sign knowledge questions correctly answered
    - **Rationale:** This outcome aggregates all the danger sign knowledge questions into one score. It is consistent with our knowledge index. To standardize the presentation of our results for each domain, we reported on the summary index in the main manuscript. Moreover, to offer more granular insights, we prioritized reporting disaggregated scores in the main manuscript (i.e., individual knowledge scores for antenatal danger signs, neonatal danger signs, and postpartum danger signs).
- Share of participants for whom medical care was sought in response to ≥ 1 antenatal, postpartum, or neonatal danger sign
    - **Rationale:** This outcome aggregates all the danger sign care seeking measures into one fraction. It is consistent with our danger sign care seeking index. To standardize the presentation of our results for each domain, we reported on the summary index in the main manuscript. Moreover, to offer more granular insights, we prioritized reporting disaggregated fractions in the main manuscript (i.e., individual fractions scores for antenatal danger sign care seeking, neonatal danger sign care seeking, and postpartum danger sign care seeking).

Secondary Outcomes

- Share of participants reporting receipt of respectful maternity care during last ANC visit
- Share of participants reporting receipt of respectful care during childbirth
    - **Rationale:** We posited that the respectfulness of care that mothers received could have been influenced by Jacaranda Health's complementary nurse mentorship program (MENTORS). To ensure a focused evaluation of PROMPTS, we excluded the above two outcomes from our main manuscript.
- Share of participants reporting self-efficacy in care seeking (i.e., feeling empowered to ask providers any questions) during last ANC visit
- Share of participants reporting self-efficacy in care seeking (i.e., feeling empowered to ask providers any questions) during pregnancy and childbirth
- Share of participants reporting use of antenatal supplements containing iron and folic acid during pregnancy
    - **Rationale:** None of the above three outcomes fit squarely into one of our six analytical domains. To focus our analysis and ensure an organized presentation of results, we excluded the above three outcomes from our main manuscript.
