# Supplementary material for: Impact evaluation of a digital health platform empowering Kenyan women across the pregnancy-postpartum care continuum: A cluster randomized controlled trial
Source: PLoS Med. 2025 Feb 3;22(2):e1004527. doi: 10.1371/journal.pmed.1004527 (PMC11835334; doi:10.1371/journal.pmed.1004527)
Supplement: S3 Text — (PDF) [file pmed.1004527.s006.pdf]

S3 Text. Detailed Description Of Knowledge, Birth Preparedness, And Danger Sign Care Seeking Outcome Measures

Knowledge Domain

- Knowledge of labor signs was compared against a list of 7 labor signs, including changes in discharge, contractions, heavy bleeding, lower abdominal pain, lower back pain, urgency to go to the toilet, and water breaking.
- Danger sign knowledge questions inquired whether a participant would seek immediate medical care or watchfully wait in response to several potentially severe symptoms. Specifically, 6 antenatal symptoms (back pain, blurred vision, decreased fetal movement, heartburn, nausea/vomiting, and vaginal bleeding), 3 postpartum symptoms (blurred vision, chest pain, and vaginal bleeding), and 3 neonatal symptoms (fever, yellow eyes, and yellow/thin stool) were assessed.

Birth Preparedness Domain

- The total number of items reported to have been completed in preparation for childbirth was compared against a list of eight items, including asking family or friends to help with childcare, buying baby clothes, choosing a hospital, discussing a birth plan, packing a bag, planning transportation, purchasing insurance, and saving money.

Danger Sign Care Seeking Domain

- Participants were asked whether they experienced any of:
    - 10 antenatal danger signs: blurred vision, breathing difficulty, contractions before 37 weeks, convulsions/loss of consciousness, decreased/absent fetal movements, fever, leaking of fluid before 37 weeks, severe abdominal pain, severe headache, and vaginal bleeding/discharge with foul odor.
    - 9 postpartum danger signs: blurred vision, breathing difficulty, calf pain/redness/swelling, chest pain, convulsions/loss of consciousness, fever, heavy/suddenly increased vaginal bleeding, severe abdominal pain, and severe headache.
    - 9 neonatal danger signs: convulsions/fits, fast or difficult breathing, fever, hypothermia, inability to feed/poor feeding, regurgitating with each feeding, umbilical redness/drainage, weakness/lethargy, and yellow eyes/soles of extremities.
