# Supplementary material for: Impact evaluation of a digital health platform empowering Kenyan women across the pregnancy-postpartum care continuum: A cluster randomized controlled trial
Source: PLoS Med. 2025 Feb 3;22(2):e1004527. doi: 10.1371/journal.pmed.1004527 (PMC11835334; doi:10.1371/journal.pmed.1004527)
Supplement: S1 Table — (PDF) [file pmed.1004527.s007.pdf]

S1 Table. Pre-Registered vs. Exploratory Outcomes Within Each Outcome Domain

Outcome	Pre-Registered		Exploratory	Domain
	Primary	Secondary		
# Signs of labor listed without prompting		X		Knowledge
Share of antenatal danger sign knowledge questions correctly answered		X		
Share of postpartum danger sign knowledge questions correctly answered		X		
Share of neonatal danger sign knowledge questions correctly answered		X		
Total # items done in preparation for childbirth		X		Birth Preparedness
Mother had plan to breastfeed within one hour of childbirth ^		X		
Late arrival at facility for childbirth (within two hours) ^			X	
Total # ANC visits attended	X			Routine Care Seeking
At least one PNC visit attended by mother within six weeks of childbirth during which own health was discussed ^	X			
Received at least the national-guideline-recommended # ANC visits ^			X	
Received at least the national-guideline-recommended # PNC visits ^			X	
Childbirth occurred in hospital or other formal health center ^		X		
Medical advice or treatment sought during prior month of pregnancy ^		X		
Medical advice or treatment sought for mother's health postpartum ^		X		
Medical advice or treatment sought for newborn's health postpartum ^		X		
Medical care sought for mother in response to ≥1 antenatal danger sign ^		X		Danger Sign Care Seeking
Medical care sought for mother in response to ≥1 postpartum danger sign ^		X		
Medical care sought for newborn in response to ≥1 neonatal danger sign ^		X		
Newborn exclusively breastfed by mother ^		X		Newborn Care
Newborn always put to sleep through the night on their back ^		X		
Newborn sung/talked to by mother many times over past 24 hours ^		X		
Mother's health discussed with a provider during at least one PNC visit ^			X	Postpartum Care Content
Provider conducted physical exam for mother during at least one PNC visit ^			X	
Provider discussed family planning with mother during at least one PNC visit ^			X	

Provider offered mother cervical cancer screening during at least one PNC visit ^			X	
Provider conducted physical exam for newborn during at least one PNC visit ^			X	
Provider provided immunization for newborn during at least one PNC visit ^			X	

Abbreviations: ANC, antenatal care; PNC; postnatal care

^ Indicator variable denoting the share of participants for whom the respective outcome was present

Notes: The table above categorizes each outcome reported on in the main text into one of three group: pre-registered primary, pre-registered secondary, or exploratory. Pre-registered primary and secondary outcomes refer to measures that we pre-registered on the United States Clinical Trials Registry (NCT05110521) and the American Economic Association's RCT Registry (AEARCTR-0008449). Exploratory outcomes refer to measures that were not pre-registered but that were strongly connected to the intervention's theory of change.
