# Supplementary material for: Impact evaluation of a digital health platform empowering Kenyan women across the pregnancy-postpartum care continuum: A cluster randomized controlled trial
Source: PLoS Med. 2025 Feb 3;22(2):e1004527. doi: 10.1371/journal.pmed.1004527 (PMC11835334; doi:10.1371/journal.pmed.1004527)
Supplement: S2 Table — (PDF) [file pmed.1004527.s008.pdf]

S2 Table. Comparison of Unadjusted and Holm-Bonferroni-Adjusted P-Values for Index Component Measures

Domain	Outcome	Unadjusted P-Value	Holm-Bonferroni-Adjusted P-Value
Knowledge	# Signs of labor listed without prompting	P < 0.001	P_{HB} < 0.001
	Share of antenatal danger sign knowledge questions correctly answered	P < 0.001	P_{HB} < 0.001
	Share of postpartum danger sign knowledge questions correctly answered	P = 0.736	P_{HB} = 0.736
	Share of neonatal danger sign knowledge questions correctly answered	P = 0.146	P_{HB} = 0.292
Birth Preparedness	Total # items done in preparation for childbirth	P = 0.003	P_{HB} = 0.010
	Mother had plan to breastfeed within one hour of childbirth ^	P = 0.666	P_{HB} = 0.666
	**Late arrival at facility for childbirth (within two hours) ^**	**P = 0.036**	**P_{HB} = 0.072**
Routine Care Seeking	Total # ANC visits attended	P = 0.161	P_{HB} = 0.644
	**At least one PNC visit attended by mother within six weeks of childbirth during which own health was discussed ^**	**P = 0.023**	**P_{HB} = 0.160**
	**Received at least the national-guideline-recommended # ANC visits ^**	**P = 0.023**	**P_{HB} = 0.160**
	Received at least the national-guideline-recommended # PNC visits ^	P < 0.001	P_{HB} = 0.001
	Childbirth occurred in hospital or other formal health center ^	P = 0.104	P_{HB} = 0.521
	Medical advice or treatment sought during prior month of pregnancy ^	P = 0.248	P_{HB} = 0.745
	Medical advice or treatment sought for mother's health postpartum ^	P = 0.396	P_{HB} = 0.791
	Medical advice or treatment sought for newborn's health postpartum ^	P = 0.864	P_{HB} = 0.864
Danger Sign Care Seeking	Medical care sought for mother in response to ≥1 antenatal danger sign ^	P = 0.079	P_{HB} = 0.157
	Medical care sought for mother in response to ≥1 postpartum danger sign ^	P = 0.015	P_{HB} = 0.046
	Medical care sought for newborn in response to ≥1 neonatal danger sign ^	P = 0.361	P_{HB} = 0.361
Newborn Care	Newborn exclusively breastfed by mother ^	P = 0.139	P = 0.139
	Newborn always put to sleep through the night on their back ^	P < 0.001	P_{HB} < 0.001
	Newborn sung/talked to by mother many times over past 24 hours ^	P < 0.001	P_{HB} < 0.001
Postpartum Care Content	**Mother's health discussed with a provider during at least one PNC visit ^**	**P = 0.028**	**P_{HB} = 0.098**
	**Provider conducted physical exam for mother during at least one PNC visit ^**	**P = 0.010**	**P_{HB} = 0.060**

	**Provider discussed family planning with mother during at least one PNC visit ^**	**P = 0.024**	**P_{HB} = 0.098**
	Provider offered mother cervical cancer screening during at least one PNC visit ^	P = 0.723	P_{HB} = 0.950
	**Provider conducted physical exam for newborn during at least one PNC visit ^**	**P = 0.017**	**P_{HB} = 0.085**
	Provider provided immunization for newborn during at least one PNC visit ^	P = 0.475	P_{HB} = 0.950

Abbreviations: ANC, antenatal care; PNC; postnatal care

^ Indicator variable denoting the share of participants for whom the respective outcome was present

Notes: P-values correspond to estimates from adjusted regression specifications. Bolding denotes outcomes for which Holm-Bonferroni adjustment alters statistical significance at the 5% level.
