# Supplementary material for: Impact evaluation of a digital health platform empowering Kenyan women across the pregnancy-postpartum care continuum: A cluster randomized controlled trial
Source: PLoS Med. 2025 Feb 3;22(2):e1004527. doi: 10.1371/journal.pmed.1004527 (PMC11835334; doi:10.1371/journal.pmed.1004527)
Supplement: S3 Table — (PDF) [file pmed.1004527.s009.pdf]

S3 Table. Attrition in Baseline Study Sample During Antenatal and Postpartum Follow-Up

Attrition Variable	Control	Treated	p-value of Difference [a]
Attrition from Baseline to Antenatal Follow-Up [b]	123 / 1813 (6.8%)	156 / 1865 (8.4%)	0.183
Attrition from Baseline to Postpartum Follow-Up [c]	321 / 2997 (10.7%)	298 / 3131 (9.5%)	0.236

[a] p-values from individual-level regressions adjusted for a treatment indicator and recruitment-facility-level normal vaginal birth volume tertile at baseline.
[b] Attrition among targeted and eligible participants at antenatal follow-up.
[c] Attrition among targeted and eligible participants at postpartum follow-up.
