# Supplementary material for: Impact evaluation of a digital health platform empowering Kenyan women across the pregnancy-postpartum care continuum: A cluster randomized controlled trial
Source: PLoS Med. 2025 Feb 3;22(2):e1004527. doi: 10.1371/journal.pmed.1004527 (PMC11835334; doi:10.1371/journal.pmed.1004527)
Supplement: S4 Table — (PDF) [file pmed.1004527.s010.pdf]

S4 Table. Baseline Characteristics of Eligible and Consented Cohort that Completed Antenatal Follow-Up

Baseline Characteristic	Control (N = 1,690)	Treated (N = 1,709)
Age (years)	26.05 (5.87)	26.24 (5.77)
Completed secondary school education or higher ^	65.9% (1,114/1,690)	68.5% (1,170/1,708)
Ability to read Kiswahili or English without difficulty ^	94.4% (1,596/1,690)	96.1% (1,643/1,709)
Married or living together ^	82.6% (1,396/1,690)	83.7% (1,431/1,709)
Size of household	3.93 (2.03)	3.58 (1.83)
Landowner ^	47.9% (809/1,689)	38.2% (652/1,706)
Access to an improved source of drinking water (e.g., piped water) ^	68.7% (1,161/1,690)	71.8% (1,227/1,709)
Access to an improved sanitation facility (e.g., flush toilet) ^	97.6% (1,650/1,690)	98.2% (1,679/1,709)
Access to a motor vehicle for travel to hospital ^	69.8% (1,180/1,690)	68.1% (1,163/1,709)
Time to travel from home to health facility (minutes)	24.30 (18.80)	23.10 (18.49)
Worked for pay in last week ^	24.6% (416/1,690)	25.4% (434/1,709)
Easy access to KES 2,000 if treatment for illness needed in household ^	27.8% (467/1,678)	28.9% (492/1,703)
Access to own mobile phone ^	90.0% (1,521/1,690)	93.3% (1,594/1,709)
Frequent/daily use of mobile phone to send text messages ^	34.2% (578/1,690)	40.1% (686/1,709)
Previously received text message(s) offering pregnancy advice from county ^	3.5% (59/1,686)	4.9% (83/1,705)
Gestational age (weeks)	27.18 (5.19)	27.09 (5.12)
Received prior ANC for current pregnancy ^	75.1% (1,269/1,690)	74.3% (1,269/1,709)
# ANC visits for current pregnancy	1.54 (1.32)	1.49 (1.29)
Fraction of knowledge questions answered correctly	0.67 (0.18)	0.69 (0.18)
Current pregnancy high-risk (e.g., due to hypertension, diabetes) ^	22.1% (367/1,662)	17.5% (298/1,704)
# Total pregnancies, including current pregnancy	2.42 (1.46)	2.28 (1.41)
Prior pregnancy high-risk (e.g., complicated by pre-eclampsia, postpartum hemorrhage) ^	39.9% (452/1,134)	44.5% (485/1,089)
PHQ-2 score	1.56 (1.60)	1.61 (1.60)

Abbreviations: ANC, antenatal care; KES, Kenyan Shilling; PHQ-2, Patient Health Questionnaire-2

^ Indicator variable denoting the % of participants for whom the respective characteristic was present

Notes: Continuous variables summarized by their sample mean and standard deviation: mean (SD); binary variables summarized by their sample mean as a %, with the respective fraction of participants.
