# Supplementary material for: Impact evaluation of a digital health platform empowering Kenyan women across the pregnancy-postpartum care continuum: A cluster randomized controlled trial
Source: PLoS Med. 2025 Feb 3;22(2):e1004527. doi: 10.1371/journal.pmed.1004527 (PMC11835334; doi:10.1371/journal.pmed.1004527)
Supplement: S5 Table — (PDF) [file pmed.1004527.s011.pdf]

S5 Table. Baseline Characteristics of Eligible and Consented Cohort that Completed Postpartum Follow-Up

Baseline Characteristic	Control (N = 2,676)	Treated (N = 2,833)
Age (years)	26.08 (5.91)	26.37 (5.80)
Completed secondary school education or higher ^	64.6% (1,728/2,676)	67.6% (1,913/2,832)
Ability to read Kiswahili or English without difficulty ^	95.1% (2,546/2,676)	96.5% (2,734/2,833)
Married or living together ^	81.9% (2,192/2,676)	82.9% (2,347/2,833)
Size of household	3.99 (2.05)	3.61 (1.84)
Landowner ^	47.8% (1,275/2,670)	37.4% (1,085/2,830)
Access to an improved source of drinking water (e.g., piped water) ^	68.8% (1,840/2,676)	70.8% (2,005/2,833)
Access to an improved sanitation facility (e.g., flush toilet) ^	97.1% (2,599/2,676)	98.3% (2,785/2,833)
Access to a motor vehicle for travel to hospital ^	70.1% (1,877/2,676)	69.3% (1,964/2,833)
Time to travel from home to health facility (minutes)	24.33 (18.26)	22.94 (17.57)
Worked for pay in last week ^	22.3% (597/2,676)	22.9% (650/2,833)
Easy access to KES 2,000 if treatment for illness needed in household ^	27.6% (730/2,649)	28.2% (797/2,823)
Access to own mobile phone ^	89.3% (2,390/2,676)	92.1% (2,610/2,833)
Frequent/daily use of mobile phone to send text messages ^	33.6% (899/2,676)	40.7% (1,153/2,833)
Previously received text message(s) offering pregnancy advice from county ^	4.2% (113/2,668)	6.3% (178/2,825)
Gestational age (weeks)	29.75 (6.17)	29.83 (6.14)
Received prior ANC for current pregnancy ^	81.7% (2,187/2,676)	81.4% (2,306/2,833)
# ANC visits for current pregnancy	1.93 (1.47)	2.00 (1.56)
Fraction of knowledge questions answered correctly	0.67 (0.18)	0.69 (0.18)
Current pregnancy high-risk (e.g., due to hypertension, diabetes) ^	23.7% (626/2,644)	19.7% (557/2,826)
# Total pregnancies, including current pregnancy	2.43 (1.48)	2.30 (1.39)
Prior pregnancy high-risk (e.g., complicated by pre-eclampsia, postpartum hemorrhage) ^	40.5% (727/1,794)	44.0% (813/1,849)
PHQ-2 score	1.50 (1.57)	1.62 (1.60)

Abbreviations: ANC, antenatal care; KES, Kenyan Shilling; PHQ-2, Patient Health Questionnaire-2
^ Indicator variable denoting the % of participants for whom the respective characteristic was present
Notes: Continuous variables summarized by their sample mean and standard deviation: mean (SD); binary variables summarized by their sample mean as a %, with the respective fraction of participants.
