# Supplementary material for: Impact evaluation of a digital health platform empowering Kenyan women across the pregnancy-postpartum care continuum: A cluster randomized controlled trial
Source: PLoS Med. 2025 Feb 3;22(2):e1004527. doi: 10.1371/journal.pmed.1004527 (PMC11835334; doi:10.1371/journal.pmed.1004527)
Supplement: S6 Table — (PDF) [file pmed.1004527.s012.pdf]

S6 Table. Intervention Impact on Additional Pre-Registered Outcomes

Outcome	Control Mean	Treatment Mean	Unadjusted Effect [a]	Adjusted Effect [b]
Share of antenatal, postpartum, and neonatal danger sign knowledge questions correctly answered [c,d,e]	0.66	0.68	0.02 **<br>95% CI: (0.00, 0.03)<br>P = 0.009	0.01 **<br>95% CI: (0.01, 0.02)<br>P = 0.001
Medical care sought in response to ≥1 antenatal, postpartum, or neonatal danger sign [c,d,e,^]	0.51	0.53	0.02<br>95% CI: (-0.02, 0.05)<br>P = 0.379	0.02<br>95% CI: (-0.01, 0.05)<br>P = 0.220
Receipt of respectful maternity care during last ANC visit [^]	0.82	0.85	0.03<br>95% CI: (0.00, 0.06)<br>P = 0.056	0.02 *<br>95% CI: (0.00, 0.04)<br>P = 0.045
Receipt of respectful care during childbirth [^]	0.91	0.91	0.00<br>95% CI: (-0.02, 0.02)<br>P = 0.936	0.00<br>95% CI: (-0.01, 0.01)<br>P = 0.823
Self-efficacy in care seeking (i.e., participant felt empowered to ask providers any questions) during last ANC visit [^]	0.67	0.69	0.02<br>95% CI: (-0.02, 0.07)<br>P = 0.273	0.01<br>95% CI: (-0.02, 0.04)<br>P = 0.383
Self-efficacy in care seeking (i.e., participant felt empowered to ask providers any questions) during pregnancy and childbirth [^]	0.81	0.83	0.02<br>95% CI: (-0.01, 0.05)<br>P = 0.177	0.02<br>95% CI: (-0.01, 0.04)<br>P = 0.153
Use of antenatal supplements containing iron and folic acid during pregnancy [^]	0.83	0.83	0.01<br>95% CI: (-0.05, 0.07)<br>P = 0.827	0.01<br>95% CI: (-0.04, 0.06)<br>P = 0.595

* P < 0.05 ** P < 0.01 *** P < 0.001

Abbreviations: ANC, antenatal care

[^] Indicator variable denoting the share of participants for whom the respective outcome was present

[a] The unadjusted model only includes covariates that account for the randomization procedure (e.g., recruitment facility's baseline normal vaginal birth volume tertile).

[b] The adjusted model adds baseline individual- and recruitment-facility-level covariates, including maternal age, gestational age, an indicator for first pregnancy, an indicator for secondary school attainment, an indicator for adequate ANC, an indicator for previous receipt of short message services (SMS) messages from one's county offering pregnancy advice, facility level, and facility county.

[c] See S3 Text for antenatal danger signs assessed.

[d] See S3 Text for postpartum danger signs assessed.

[e] See S3 Text for neonatal danger signs assessed.
