# Supplementary material for: Impact evaluation of a digital health platform empowering Kenyan women across the pregnancy-postpartum care continuum: A cluster randomized controlled trial
Source: PLoS Med. 2025 Feb 3;22(2):e1004527. doi: 10.1371/journal.pmed.1004527 (PMC11835334; doi:10.1371/journal.pmed.1004527)
Supplement: Attachment — Submitted filename: PLOS Medicine Revised Manuscript - Point-by-Point Response.docx [file pmed.1004527.s013.docx]

**Date:** 13 November 2024

**Manuscript Number:** PMEDICINE-D-24-01960R1

**Article Title:** “Empowering patients across the maternal-newborn care continuum: A cluster randomized controlled trial testing a digital health platform in Kenya”

**Corresponding Author:** Rajet Vatsa ([rajet_vatsa@hms.harvard.edu](mailto:rajet_vatsa@hms.harvard.edu))

Dear *PLOS Medicine* Editorial and Review Team:

Thank you so much for your thoughtful review of our team’s research article. We acknowledge how selective and rigorous a publishing outlet *PLOS Medicine* is and thus appreciate each of the reviewers’ suggestions to strengthen our work. In our enclosed revision, we have attempted to address each comment provided and describe our changes below (see next page) in a point-by-point manner. Page and line numbers refer to the manuscript version with no markup.

Overall, the major changes that we have made are as follows:

1. **Clarifying Outcomes Assessed:** Though we organize our outcomes into six domains, each domain is comprised of component measures that we also report on. In our revision, we have explicitly indicated whether each component outcome was pre-registered or exploratory. We have added a supplementary table which delineates pre-registered primary vs. pre-registered secondary vs. exploratory index component measures. In addition, we have added a section to the supplement that lists and defines pre-registered outcomes that we do not report on in the main manuscript, with corresponding rationales; results for these outcomes are provided in an additional supplementary table.
2. **Improved Adjustments for Multiple Comparisons:** In our initial submission, our only statistical approach to account for multiple hypothesis testing was aggregating pre-registered and exploratory outcomes into inverse-covariance-weighted summary indices. In our revision, we defend this method, which is a common way to aggregate outcomes and limit the number of hypotheses tested. At the same time, we have taken additional steps to account for multiplicity. Given that we have multiple domains, we report Holm-Bonferroni-adjusted p-values for each summary index to control the family-wise error rate (FWER) across domains. As for our index components, we have provided a side-by-side comparison of unadjusted and Holm-Bonferroni-adjusted p-values in a new supplementary table. The adjusted p-values control the FWER for each domain, and we assess the robustness of our findings to these adjustments.
3. **Clarifying Power Calculations:** We have also clarified how our power calculations were done, including the focus on two primary outcomes for which we had administrative data. In our revision, we have also clarified how we derived our enrollment goal and have updated our numbers to reflect the underlying assumptions of our power calculations.
4. **Improved Language Regarding our Study Population:** Our initial submission contained conflicting language regarding our study population: patients vs. healthy pregnant and postpartum women. Our target population included all eligible pregnant and postpartum women, independent of their health status. We have streamlined our language throughout the manuscript to clarify this.
5. **Clarifying the Guidelines Underlying our Outcomes:** In our initial submission, there was ambiguity regarding what constituted guideline-based care. In our revision, we have clarified that our assessment of routine antenatal and postnatal care seeking is based on Kenya’s National Guidelines for Quality Obstetrics and Perinatal Care. These guidelines are still in place in Kenya and served as the basis for PROMPTS message content around routine care seeking. We have eliminated all references to potentially conflicting or outdated World Health Organization guidelines.

Altogether, we believe the changes we have made have strengthened our manuscript in terms of organization, clarity, and statistical rigor. Again, thank you for all the feedback to help us get to this stage. Please do not hesitate to reach out if we can clarify any of our responses/revisions. We appreciate your continued time and consideration and look forward to hearing back.

Sincerely,

Rajet Vatsa, on behalf of the authorship team

Harvard University PhD Program in Health Policy | [Rajet_Vatsa@hms.harvard.edu](mailto:Rajet_Vatsa@hms.harvard.edu) | +1-480-747-4380

**Point-by-Point Response to Reviewer and Editorial Comments:**

Reviewer 1

1. There seems to be a lack of connection between the power calculation and the choice of outcomes. The power calculation (lines 335-343) mentions a 6.5 percentage point increase in rate of postnatal care receipt whereas the main outcomes are the various indices. It is also not entirely clear how the target of 4,800 women was arrived at. According to my calculation, with 40 clusters, an ICC of 0.01, a base proportion of 61% and an absolute increase of 6.5 percentage point, one needs an average cluster size of 74 participants i.e. a total sample size of 2,960 (with 80% power and 2-sided alpha of 5%). Please clarify/correct.

Thank you for raising this important point and for alerting us to potential errors in the numbers we report. We focused our power calculations on two pre-registered primary outcomes for which we had available administrative data prior to our study: 1) the rate of postnatal care receipt within six weeks of childbirth (during which the mother’s own health was discussed); and 2) the total number of antenatal care visits. The enrollment goal of 4,800 women (roughly 120 women per study facility) accounted for the assumption that ≥80% of women in treatment facilities would enroll in PROMPTS and that ≥60% of those women would be successfully engaged through endline: $120 \times0.8 \times0.6\approx58$ assessable women per study facility. We have clarified this in the text and have updated the numbers (Pages 19-20; Lines 405-416).

2. It is not clear what the primary outcome is and, if multiple outcomes were considered at the same (primary) level, how multiplicity was accounted for. Given the number of outcomes at the same level, it would make sense to apply a method to control for multiplicity. I would suggest Holm-Bonferroni or Holm-Sidak correction. This could potentially be applied first across all summary indices (one family), then across sub-indices within each category (e.g. knowledge category = 4 sub-indices)

We appreciate each of these suggestions. One of the challenges of evaluating the PROMPTS platform is that it attempts to influence a variety of outcome domains and does not have a singular primary outcome. The primary pre-registered outcomes of our trial were the number of antenatal care visits, the share of mothers receiving postnatal care within six weeks of childbirth, danger sign knowledge, the rate of danger sign care seeking, quality of routine maternity care, quality of care for management of neonatal resuscitation, and provider knowledge of obstetric and newborn care. We are analyzing the latter three outcomes in other work that evaluates the impact of Jacaranda Health’s complementary nurse mentorship program (MENTORS).

In recognition of the large number of outcomes, we have organized our analysis within six domains and use summary indices to reduce the risk of false discovery from multiple hypothesis testing. This approach is common in evaluations of programs with multiple objectives (e.g., [Anderson 2008](https://are.berkeley.edu/~mlanderson/pdf/Anderson%202008a.pdf); [Finkelstein et al. 2013](https://pmc.ncbi.nlm.nih.gov/articles/PMC3535298/)). The inverse-covariance-weighted nature of our indices also has the benefit of down-weighting highly correlated components, thereby emphasizing index components that offer unique information. We have emphasized this in the Methods > Outcomes section (Page 13; Lines 306-315).

Nonetheless, we recognize and agree that additional steps could be taken to further reduce the risks of testing multiple hypotheses. To account for our multiple domains and control the family-wise error rate (FWER) across domains at 0.05, we report Holm-Bonferroni-adjusted p-values for each summary index (described on Page 19; Lines 398-399). Moreover, while we report unadjusted p-values for the index components in the main manuscript (given our other steps to control for multiplicity), we provide a side-by-side comparison of unadjusted and Holm-Bonferroni-adjusted p-values in a new supplementary table (S7 Table; Pages 52-53). The latter control the FWER for each domain at 0.05 (described on Page 19; Lines 400-404). We assess the robustness of our findings for individual index components to this adjustment in the Results > Sensitivity Analysis section (Page 31; Lines 616-626).

3. Please clarify whether a pre-specified statistical analysis plan was developed/published before unblinding. If so, I would strongly recommend its inclusion as supplemental material.

While we pre-registered our evaluation on the United States Clinical Trials Registry (NCT05110521) and the American Economic Association’s RCT Registry (AEARCTR-0008449), as reported in the manuscript, we did not have a separate pre-specified statistical analysis plan. We pre-specified primary and secondary outcome measures in the trial registries, which served as the basis for the domains that we report on. We have attempted to clearly denote pre-registered vs. exploratory component measures throughout the text. We have also added a supplementary table (S4 Table; Pages 48-49), which delineates pre-registered primary vs. pre-registered secondary vs. exploratory index component measures. In addition, we have added a section to the supplement (S5 Text; Page 50), listing pre-registered outcomes that we do not report on in the main manuscript, along with corresponding rationales.

4. Abstract: I would suggest reporting the p-value for the main composite indices in addition to 95% CI to help assess the strength of evidence.

We appreciate this suggestion and have added p-values for the summary indices in addition to the 95% confidence intervals in the abstract (Page 2; Lines 51-58) and main text.

5. Please clarify the process, if any, used to ensure that facilities from different study arms were at least 10 kilometres apart. I understand that randomisation was stratified by tertile of monthly volume; however, there is no mention of stratification by geographical location or that randomisation was constrained by distance between facilities. Please also consider adding details of who was responsible for randomisation including allocation concealment procedures (at what point were facilities informed of their random allocation?).

Thank you for these important clarifying questions. Indeed, we only stratified our randomization by tertile of monthly volume, not by geographical location/inter-facility distance. However, steps were taken a priori to ensure that no two facilities were too close to one another. Specifically, candidate facility locations were reviewed with the team at Jacaranda Health, and in select cases where two facilities were near one another (e.g., in the same sub-county), the facility with the lower level or volume was excluded. Thus, the randomized set of 40 facilities represented 40 distinct sub-counties. We have clarified this in the main text in the Methods > Randomization and Masking section (Pages 10-11; Lines 253-258).

Moreover, after running the randomization algorithm in Stata (performed by the Harvard research team), we again examined the facility locations visually on a map to check for facilities that were assigned to different arms but that were located close to each other. On detailed review with the Jacaranda Health team, we confirmed that facilities from different arms were at least 10 kilometers apart; even those that were geographically “close” together could be quite far away in terms of transit time. We have clarified this in S3 Text (Page 47).

Finally, regarding the random allocation concealment procedure, assignment was not concealed due to the nature of the intervention (e.g., recruitment of patients at ANC clinics for PROMPTS enrollment). Facilities were informed about their allocation before study activities commenced. We have clarified this in the main text in the Methods > Randomization and Masking section (Page 11; Lines 260-261).

6. For recruitment of pregnant women, please clarify whether all eligible women were approached or if some selection process was applied. According to Figure 2, 3,662 and 3,622 women were assessed for participation in the treatment and control facilities respectively. Please provide details around the selection of women "assessed for participation". For example, were all pregnant women who came to the facility assessed?

We have clarified this in the main text in the Methods > Study Design, Intervention, and Participants > Participant Recruitment and Eligibility section (Page 10; Lines 249-250). In short, at baseline, all women who arrived at the facility for antenatal care were assessed for eligibility and consent, subject to enumerator availability. No other selection process was applied.

7. I would suggest adding a table defining all pre-defined and exploratory indices to supplement the text included on lines 278 - 313 of the manuscript.

Thank you for this valuable suggestion. We have added a supplementary table (S4 Table; Pages 48-49), which delineates all pre-registered primary vs. pre-registered secondary vs. exploratory index component measures. In addition, we have added a section to the supplement (S5 Text; Page 50), listing/defining pre-registered outcomes that we do not report on in the main manuscript, along with corresponding rationales.

8. Lines 331-334 indicates that standard errors were clustered by enrolment facility. Was this done by using Generalised Estimating Equations? Please confirm / clarify in the manuscript.

We obtained clustered standard errors by first estimating ordinary least squares regressions and then using clustered sandwich estimators to adjust our inferences, as detailed in Zeileis, Köll, and Graham (2020). We have clarified this in the manuscript in the Methods > Statistical Analysis section (Page 19; Lines 395-396).

9. Please add the Treatment mean to Table 4 and Table 5.

We have added a Treatment Mean column next to the Control Mean column in Table 4 (Page 24) and Table 5 (Pages 26-27).

10. I find that Figure 4 lacks clarity. First, it is not clear to me why these 3 metrics were picked out of all possible indices. Second, the spacing between bars does not seem to be consistent. It is particularly visible on the top left panel where the number and alignment of bars between 4 and 8 seems odd. Third, I am not convinced that the current display provides enough evidence of an increase in timing between arrival and childbirth as stated in the footnote. Please consider a different display and/or a statistical test to assess this hypothesis.

Thank you for this valuable feedback. We have streamlined this figure (Page 29) in a couple of key ways: 1) we have removed the panel on the timing of facility arrival (an exploratory analysis) to restrict the focus to antenatal and postnatal care volume, which are related to two of our primary, pre-registered trial outcomes, and show an interesting pattern: increases in ANC and PNC utilization around the guideline-recommended thresholds; and 2) we adjusted the spacing between histogram bars in the remaining panels to ensure consistency and alignment with axis labels.

11. Please consider present all pre-specified indices in a single table. One option could be to combine table 4 and 5 and/or having a separate table for exploratory outcomes i.e. by combining 4.

We appreciate this suggestion and did consider possible designs that would enable including all outcomes. However, ultimately, we elected to keep things organized as is, to not include too many results in one table. We hope the other changes addressed above alleviate the confusion around the outcomes reported on and our rationale to organize our findings under domains/families of hypotheses.

Reviewer 2

12. What was the participants response/feedback on receiving frequent messages [10-40 text messages as mentioned in the manuscript]?

While it would have been interesting to hear the qualitative perspectives of participants, we did not conduct qualitative assessments with participants in our study to assess their feedback on the messages they received. However, Jacaranda Health has conducted significant formative work in this area, including testing the time of day that users prefer to read and engage with messages and assessing the optimal message frequency. We have added a brief comment about this in the Methods > Study Design, Intervention, and Participants > Intervention section (Page 9; Lines 226-229).

In addition, Jacaranda Health features a number of testimonials on their website from participants whose pregnancies have been benefited by PROMPTS. Select patient stories can be found here: [Esther’s Story](https://jacarandahealth.org/driving-positive-breastfeeding-behaviors-amongst-new-moms-via-sms/); [Faith’s Story](https://jacarandahealth.org/how-automation-is-helping-connect-at-risk-mothers-to-emergency-transport-and-quality-care/); [Violet’s Story](https://jacarandahealth.org/finding-a-voice-through-our-digital-prompts-platform/). We do not present these findings in our manuscript as they were independent from our study.

13. What about those pregnant women who could not read/were illiterate? were these only text message or audio messages were sent too?

This is an important point; thank you for bringing it up. While the latest version of the PROMPTS platform offers audio support for low-literacy or sight-impaired mothers, the version that was rolled out at the time of our study only featured text-based campaigns. We have clarified this in the manuscript in the Methods > Study Design, Intervention, and Participants > Intervention section (Page 10; Lines 240-241).

It is also worth emphasizing that the content that participants receive has been rigorously tested by Jacaranda Health to ensure broad accessibility; before new content is deployed, it is tested for comprehension among those with a basic primary education, in both urban and rural settings.

In our sample, while there were participants at baseline who could not read either English of Kiswahili, we did not examine their outcomes separately, as they constituted a small sample (138/6,139; 2.2%).

14. Duration and dates of study should be included

Thank you for this suggestion. We have included the specific date ranges for baseline data collection (timing of study enrollment), antenatal follow up, and postpartum follow up in the Methods > Procedures > Data Collection section (Page 13; Lines 294-296).

Reviewer 3

15. L72: "Low- and lower-middle income settings" vs. "low- and middle-income settings in L87 and L128": Are they the same or different?

Thank you for noting this discrepancy in our language. We have updated the language in our opening paragraph to say “low- and middle-income settings” as well (Page 4; Line 106).

16. L82: In this line, please specify what it means by "the recommended number of ANC visits. Ref. 39 shows the WHO's recommendation, which recommended the "ANC models with a minimum of eight contacts." Has Kenya followed this model? This article does not seem to follow the WHO's recommendation.

Thank you for raising this point. Indeed, the WHO’s latest recommendation specifies a minimum of eight ANC contacts. Prior to this recommendation from 2016, the WHO outlined a “focused ANC” model that called for a minimum of four personalized and comprehensive ANC visits. Kenya’s [National Guidelines for Quality Obstetrics and Perinatal Care](http://guidelines.health.go.ke:8000/media/National_Guidelines_for_Quality_Obstetrics_and_Perinatal_Care.pdf) are based on the focused ANC model. Similarly, Kenya’s guidelines for recommended postnatal care follow a targeted postnatal care model, which we use as the basis for our guideline-recommended PNC outcome.

We have updated our language/references throughout the manuscript to emphasize these national guidelines, upon which the PROMPTS messages are also based (Page 17; Lines 359-361).

17. L84 and other places: Please check the reference numbers; some were not written with superscript.

We have gone through the text and ensured that all our reference numbers are formatted consistently.

18. Regarding the use of the term "patients," I have many comments.
    a. L112, L123: In these two paragraphs, "digital health tools" are much more emphasized as the tools for "patients" rather than for "healthy pregnant women and mothers," though they were also mentioned a little. It seems better to tone down this "patient" description.
    b. L139: What does "a patient-level solution" mean? Was the target population of this article "patients"?
    c. L146 and other places: All the pregnant women seem to be defined as "patients" in this article, even if some had no danger signs or symptoms such as anemia.
    d. L158~: "Patients" were used in treatment facilities. "Participants" and "women" were used in control facilities. It suggests the target population in the treatment facilities had some medical problems, and those in the control facilities had no such issues. In this article, PROMPS stands for "Promoting Mothers in Pregnancy and Postpartum Through SMS." Must all the mothers who can join PROMPS "patients"?
    e. L201: Eligibility: Must pregnant women be patients in the treatment group?
    f. L240, L244: Were they patients?
    g. L334: Not all the participants were "patients" from this description. Please reconsider the use of "patients" throughout the manuscript, including the introduction contents.

Thank you for raising all these important points. We want to be mindful and clear in our language, so appreciate the nuanced comments here. Indeed, the target population in both treatment and control facilities is pregnant and postpartum women, independent of whether they are patients (as defined by having some danger sign or symptom). We have updated our language throughout the manuscript (including in the title, abstract, introduction, figures, etc.) to better reflect this (e.g., replacing “patients” with “pregnant and postpartum women”).

19. L132: MNH nonprofit organization?

Thank you for this suggestion. We have updated our phrasing accordingly (Page 6; Line 171).

20. L136-138: What was the coverage of health facilities and individuals (pregnant women and mothers?) when this study started? Were both coverages increased without having the evidence shown in this article? If so, what is the value of this article?

Thank you for raising this question. We agree that the coverage of health facilities and individuals at the start of our study period is more relevant to report. We have updated these statistics in the manuscript: ~950 health facilities and 750,000+ individuals by November 2021 (Page 7, Lines 173-175).

Both coverages have increased since the time of the manuscript publication. This expansion may reflect the perceived need for such programming, as well as the perceived value of the intervention by government partners. In addition, an earlier Jacaranda Health RCT (smaller scale from 2017-2018) demonstrated positive behavior changes in the postpartum period due to a similar SMS-based intervention. We include mention of this study in the last paragraph of the Introduction (Page 7, Lines 176-178) and in the Discussion section (Page 35; Lines 709-711, 720-721). Finally, Jacaranda Health saw their most rapid uptake of PROMPTS (by county governments) during the COVID-19 pandemic, when a lot of counties felt the need for digital tools to support their lack of service delivery capabilities.

Notwithstanding this increase in coverage of the program, causal evidence of the program’s impact is critical for assessment of the need to sustain the expansion and can inform efforts to invest in similar systems in other contexts. It can also identify important areas for intervention improvement; for instance, the improvements in danger sign knowledge and care seeking that we identified were quite low, despite these being stated priorities of PROMPTS. We have added a more explicit discussion of potential places for improvement in the Discussion section (Pages 32-33; Lines 658-664).

21. L140: Again, please describe "the guideline-recommended." Which guideline by who?

We have deleted the phrase “guideline-recommended” from this paragraph to avoid confusion, but we have addressed this important point in other parts of the manuscript (Page 17; Lines 359-361), as described in points 16 and 27.

22. L144: For international readers, please briefly describe Kenya's health system and related MNH policies.

We have added some additional context on Kenya’s health system (e.g., facility levels) in the Methods > Randomization and Masking section (Page 11; Lines 265-268). With respect to Kenya’s MNH policies (e.g., around recommended antenatal and postnatal care), we have elaborated on this in the Methods > Outcomes section (Page 17; Lines 359-361), as discussed in points 16 and 27.

23. L156: How did you know "the other interventions" influenced or not influenced this evaluation?

Thank you for raising this point. Ultimately, we cannot know for certain whether the provider-facing MENTORS intervention influenced the outcomes in our evaluation. We selected outcomes for our main manuscript that should not have been influenced by a provider-facing emergency obstetric care program, and 97% of the PROMPTS sample delivered in study facilities before rollout of MENTORS training was complete (as stated in the manuscript). Nevertheless, to avoid overstating, we have removed the phrasing about the influence (or lack thereof) of the MENTORS intervention from the Methods > Study Design, Intervention, and Participants section (Pages 7-8; Lines 191-198).

24. L170: In this paragraph, do participants mean patients?

We hope our clarification in point 18 addresses this comment; participants include pregnant and postpartum women, independent of whether they are patients (as defined by having some danger sign or symptom). Thank you again for prompting us to clarify our language.

25. L211: 100 Kenyan shillings. How much is it in US dollars?

At the time of our study, 100 Kenyan shillings was worth slightly less than 1 USD. We have added this information to our manuscript (Page 13; Line 297).

26. L229: Please clarify the primary and secondary outcomes in the abstract and the main text. In this place, the primary and secondary needs to be explained.

We appreciate this comment and acknowledge its importance. As discussed in point 2, one of the challenges of evaluating the PROMPTS platform is that it attempts to influence a variety of outcomes. In recognition of the large number of outcomes, we have organized our analysis within six domains. Each domain is composed of component measures, which we list/define and classify as either pre-registered or exploratory in the Methods > Outcomes section (Pages 13-18; Lines 306-376). We have attempted to clearly denote pre-registered vs. exploratory component measures throughout the text. We have also added a supplementary table (S4 Table; Pages 48-49), which delineates pre-registered primary vs. pre-registered secondary vs. exploratory index component measures. In addition, we have added a section to the supplement (S5 Text; Page 50), listing pre-registered primary and secondary outcomes that we do not report on in the main manuscript.

In our abstract, we focus on the six domains to streamline the presentation of our findings and control for multiplicity. However, in the main text/supplement, we clarify our various types of component outcomes.

27. L253: Total number of ANC and PNC visits: What is the policy about them? Again, in Ref 39 (WHO), WHO recommended eight ANC visits.

Thank you again for bringing up this important point. As mentioned in point 16, indeed, the WHO’s latest recommendation specifies a minimum of eight ANC contacts. Prior to this recommendation from 2016, the WHO outlined a “focused ANC” model that called for a minimum of four personalized and comprehensive ANC visits. Kenya’s [National Guidelines for Quality Obstetrics and Perinatal Care](http://guidelines.health.go.ke:8000/media/National_Guidelines_for_Quality_Obstetrics_and_Perinatal_Care.pdf) are based on the focused ANC model. Similarly, Kenya’s guidelines for recommended postnatal care follow a targeted postnatal care model, which we use as the basis for our guideline-recommended PNC outcome. We have updated our language/references throughout the manuscript to emphasize these national guidelines (Page 17; Lines 359-361).

28. L258: Considering stagnant neonatal mortality rates in Kenya, the first PNC (usually within 24 or 48 hours after birth) might be the most critical PNC visit. However, in this article, 1st and 2nd PNC visits and 2nd and 3rd PNC visits are considered the same. Is there data to show 1st and 2nd or 1st and 3rd, for example?

This is an important point, and we appreciate the reviewer bringing up this nuance. Unfortunately, we are unable to disentangle the timing of each PNC visit. In the postpartum follow-up survey, we did inquire about the timing of the first postpartum check, but this was not a pre-specified outcome and was somewhat imperfectly measured (e.g., not always clear whether the response provided was in days or weeks). Thus, we only report on the overall quantity of PNC visits attended by the time of postpartum follow-up.

29. L270: Were the weight of the 1st, 2nd, and 3rd PNC visits the same?

Yes, building off our prior response, each PNC visit is weighted equally. In our data, we are unable to disentangle the timing of each PNC visit. Thus, we only report on the quantity of PNC visits for each respondent overall, without placing a higher/lower weight on any specific visit (based on its timing, duration, or some other attribute).

30. L287: Each ANC visit has the best timing for it. "Adequate prenatal care" means only the number of ANC visits, and its timing was not counted in this article.

This is another great point. We did in fact account for a respondent’s gestational age at enrollment to determine whether the quantity of previous prenatal care was adequate. In other words, the indicator for adequate prenatal care was conditionally defined based on one’s gestational age. We have clarified this in the manuscript (Page 19; Lines 391-392).

31. L333: If participants do not have their own mobile phones, how did they have access to mobile phones? Were there any differences between the own-phone users and non-users?

While participants were required to have access to a mobile phone, per the study’s inclusion criteria, it could have been their own mobile phone or some close contact’s (e.g., spouse’s) mobile phone. We have clarified this in the manuscript (Page 21; Lines 447-448).

We did not include heterogeneity analyses in our manuscript to control the number of results reported, given the already high number of hypotheses evaluated. However, briefly, ~90% (5,499/6,139) of participants in our baseline sample had their own mobile phone. While the majority of outcomes were similar for own-phone users and non-own-phone users, own-phone users reported lower rates of antenatal danger signs (with correspondingly lower rates of antenatal danger sign care seeking) and demonstrated potentially associated higher levels of improvement in antenatal knowledge scores. Own-phone users also exhibited higher rates of care seeking for postpartum maternal danger signs compared to non-own-phone users.

32. L339, 340: Participants are equal to respondents?

Yes, we used these terms interchangeably. We have modified the language in the relevant paragraph to make this clearer.

33. L346: Over 85 % received the messages in the intervention group, and about 10 % received them in the control group. Were there any differences in outcomes between those who received and those who did not receive in each group? Were there any similarities in outcomes between those who received them in both groups?

This is an interesting question and something we thought about as well. Ultimately, we did not conduct such subgroup analyses in our manuscript, in part to control the number of results reported, given the already high number of hypotheses evaluated. Moreover, and perhaps more importantly, such comparisons (e.g., exposed individuals in treatment vs. control arm) would be subject to selection bias. We instead present unbiased, intention-to-treat (rather than per-protocol) estimates from our overall sample.

34. L403, L415: These paragraphs must be the highlight of this article. They can be strengthened by using more references.

Thank you for this suggestion. The paragraphs mentioned here contain a summary of the key findings from our analyses. We agree that it is important to contextualize these findings in terms of what is known from other references, which we do in the paragraphs that follow (Pages 33-36; Lines 665-729). We have also added insights from additional references in those paragraphs.

35. L412, L512: "Mere 74 cents" seems reasonable to justify the feasibility. Considering sustainability by the Kenyan government or society, is it unnecessary to consider consultancy, management, and other implementation costs for this platform?

Thank you for raising this question. The 74-cent cost that we mention includes:

- SMS costs ($0.28): Jacaranda Health pays $0.004 per message.
- Technology Infrastructure (Amazon Web Services, Enrollment Data Capture, Data Storage, PROMPTS software hosting, Helpdesk) ($0.13).
- Field Enrollment Costs ($0.15): Costs incurred by personnel to directly enroll mums. In the scenario with county cost share, they incur $0.11 of this cost.
- County Engagement/Enrollment Visits ($0.18): Centralized visits at the County and Subcounty level for training or update reports.

Thus, management and implementation costs are included in addition to the operational/technical infrastructure costs. We have clarified this in the manuscript (Page 32; Lines 645-646).

36. L413,414: The PROMPTS has been implemented at a remarkable scale in a few years. Based on which evidence was it scaled? Supposing it was scaled without the evidence of this article, what would be the value of this article?

As discussed in point 20, one potential source of evidence was an earlier Jacaranda Health RCT (smaller scale from 2017-2018), which demonstrated positive behavior changes in the postpartum period due to a similar SMS-based intervention. In addition, Jacaranda Health saw their most rapid uptake of PROMPTS (by county governments) during the COVID-19 pandemic, when a lot of counties felt the need for digital tools to support their lack of service delivery capabilities. It may also just be the case that it is easy to scale a low-cost program because participants like it, even if it doesn’t impact other outcomes.

As other counties and countries now consider a tool like PROMPTS, causal evidence of the program’s impact is critical for assessment of the need to sustain the expansion. It can also identify important areas for intervention improvement; for instance, the improvements in danger sign knowledge and care seeking that we identified were quite low, despite these being stated priorities of PROMPTS. We have added a more explicit discussion of potential places for improvement in the Discussion section (Pages 32-33; Lines 658-664).

More generally, we believe our article will serve as a valuable addition to the evidence base, with findings from an intervention implemented at scale and geared towards both pregnant and postpartum women and their newborns in a low- and middle-income setting.

37. L441: Is it relevant to use Ref 46 in this paragraph if the target population of this article was not patient in general?

Thank you for raising this clarifying question. We have removed the word “patient” from this sentence (Page 34, Line 683). Both this reference and our article focus on pregnant women and mothers broadly speaking, not on patients. The reference is accordingly still relevant, so we have kept it in the manuscript.

38. L490: In L156, it was written that "the other interventions" did not influence the outcome of this evaluation. "Jacaranda Health's complementary mentorship program" was not one of "the other interventions"?

Thank you for pointing out this discrepancy in our manuscript. Hopefully the changes we made in response to point 23 address this. Indeed, we cannot know for certain whether the provider-facing MENTORS intervention influenced the outcomes in our evaluation. Accordingly, we removed the phrasing about the influence (or lack thereof) of the MENTORS intervention from the Methods > Study Design, Intervention, and Participants section (Pages 7-8; Lines 191-198).

39. L513: "Platform's low cost" sounds like the total cost considering total management and implementation. It must be part of the cost of its implementation.

As discussed in point 35, the 74-cent cost that we mention includes the costs of SMS message delivery, backend technical infrastructure, field enrollment, and county engagement. It is a more comprehensive cost estimate than a mere estimate of the operating/management costs (Page 32; Lines 645-646).

40. L562: Inside the figure, "Treatment" was written in black letters, and "control" was written in green letters. The title of Fig. 4 was about the Treatment Arm. What does it mean by "treatment" and "control" in this figure?

Thank you for raising this clarifying question. We intended for this figure to show histograms of each outcome among individuals recruited from treatment vs. control facilities. We have updated the figure (and its title) to make this clearer (Page 29). The gray bars correspond to individuals recruited from control facilities, and the overlaid blue bars correspond to individuals recruited from treatment facilities.

41. L726: References: Some reference names were fully spelled out, and some reference names were abbreviated. Please make it consistent.

We have gone through each of our references and ensured that they are formatted consistently, including abbreviating all journal names per the NCBI database, when applicable (Pages 61-65).

General Editorial Comments

42. Please upload any figures associated with your paper as individual TIF or EPS files with 300dpi resolution at resubmission.

We have run all our figures through PACE and included them as individual TIF files with the requested resolution in our resubmission.

43. Thank you for providing a Data Available Statement. PLOS has a strong stance that requires data to be available where at all possible. Could the authors possibly be able to provide de-identified or anonymized data?

Unfortunately, we are unable to provide de-identified or anonymized data. The ethical approval under which our study data were collected (along with the corresponding participant consent forms) indicated that the data were to be accessible to only the study team and would not be transmitted elsewhere.

44. We expect all researchers with submissions to PLOS in which author-generated code underpins the findings in the manuscript to make all author-generated code available without restrictions upon publication of the work. Authors are responsible for ensuring that the code is reusable and well documented. Please make any custom code available, either as part of your data deposition or as a supplementary file. Please add a sentence to your data availability statement regarding any code used in the study, e.g. "The code used in the analysis is available from Github [URL] and archived in Zenodo [DOI link]".

We have uploaded our analysis code to a public GitHub repository: https://github.com/RVatsa96/prompts-evaluation. We have ensured that our code is reusable and well-documented. We are happy to answer any questions about our analytical pipeline.

45. Thank you for providing a completed CONSORT checklist. Please add the following statement, or similar, to the Methods: "This study is reported as per [XXXX] guideline (S1 Checklist)."

We have added this statement to the end of our first Methods sub-section (Page 9; Lines 220-222) and have accordingly rearranged our Supporting Information to include both of our CONSORT checklists as the first two supplementary exhibits (Pages 40-46).

46. At this stage, we ask that you include a short, non-technical Author Summary of your research to make findings accessible to a wide audience that includes both scientists and non-scientists. The Author Summary should immediately follow the Abstract in your revised manuscript. This text is subject to editorial change and should be distinct from the scientific abstract. Ideally each sub-heading should contain 2-3 single sentence, concise bullet points containing the most salient points from your study. In the final bullet point of 'What Do These Findings Mean?', please include the main limitations of the study in non-technical language.

We have included our Author Summary immediately following the Abstract in our revised submission (Page 3; Lines 71-103).

47. Please express the main results with 95% CIs as well as p values. When reporting p values please report as p<0.001 and where higher as the exact p value p=0.002, for example. Throughout, suggest reporting statistical information as follows to improve clarity for the reader "22% (95% CI [13%,28%]; p</=)". Please be sure to define all numerical values at first use.

We have expressed all our main results in the specified format, in both our text and tables.

48. Please provide titles and legends for all figures and tables (including those in Supporting Information files).

We have ensured that all our figures and tables, including those in Supporting Information files, have titles and legends/notes.

49. Please define all abbreviations used in each figure/table (including those in Supporting Information files).

We have gone through all our figures and tables and defined abbreviations in the respective legends/notes.

50. Please consider avoiding the use of red and green in order to make your figure more accessible to those with color blindness.

Thank you for this important suggestion. We have updated Figure 1 (Page 8), Figure 3 (Page 14), Figure 4 (Page 29), and Figure 5 (Page 30) to eliminate the use of red and green.

51. Please cite your Supporting Information as outlined here: <https://journals.plos.org/plosmedicine/s/supporting-information>

We have followed the in-text citation guidelines for our Supporting Information, as outlined in the link above.

52. PLOS uses the numbered citation (citation-sequence) method and first six authors, et al. Please ensure that journal name abbreviations match those found in the National Center for Biotechnology Information (NCBI) databases (<http://www.ncbi.nlm.nih.gov/nlmcatalog/journals>), and are appropriately formatted and capitalized. Where website addresses are cited, please include the complete URL and specify the date of access (e.g. [accessed: 12/06/2023]).

We have gone through each of our references and ensured that they are formatted consistently, including abbreviating all journal names per the NCBI database, when applicable. We have also specified the date of access whenever we provided complete website addresses (Pages 61-65).

53. Please structure the Methods section using the following sub-headings: Study design and participants, Randomization and masking, Procedures, Outcomes, Statistical analysis.

We have restructured the Methods section (Pages 7-20) using the following sub-headings, with some minor exceptions. Our first part of the Methods section offers a detailed description of our study intervention, so we label the first sub-section “Study Design, Intervention, and Participants.” Finally, we include a sub-section at the end with our “Ethics and Safety Statement.”

54. Please ensure that all prespecified outcomes (primary, secondary, and exploratory) are listed in the Methods/Outcomes section and indicate whether there are outcomes that are not presented in the current report.

We have listed all outcomes that we test in the Methods > Outcomes section (Pages 13-18; Lines 306-376), denoting those which are prespecified vs. exploratory. We have also added a supplementary table (S4 Table; Pages 48-49), which delineates pre-registered primary vs. pre-registered secondary vs. exploratory index component measures. In addition, we have added a section to the supplement (S5 Text; Page 50), listing pre-registered primary and secondary outcomes that we do not report on in the main manuscript, along with corresponding rationales. Results for these additional outcomes are provided in a new supplementary table (S12 Table; Page 60).

55. Please specify the dates (Month Day, Year) during which study enrollment and follow up occurred.

We have included the specific date ranges for baseline data collection (timing of study enrollment), antenatal follow up, and postpartum follow up in the Methods > Procedures > Data Collection section (Page 13; Lines 294-296).

56. Please include absolute numbers wherever you report percentages; eg, n/N (%)

We have added absolute numbers wherever we report percentages in our results (abstract [Page 2], main text, Table 2 [Pages 22-23], S10 Table [Pages 56-57], S11 Table [Pages 58-59]). When citing background statistics in the introduction, methods, and discussions, we simply include rough percentages.

57. In keeping with our commitment to Open Science, please include the study protocol document and analysis plan (including any amendments) as Supporting Information to be published with the manuscript if accepted.

While we did not have a prespecified analysis plan, we pre-registered our evaluation on the United States Clinical Trials Registry (NCT05110521) and the American Economic Association’s RCT Registry (AEARCTR-0008449), as reported in the manuscript. We have also included our initial trial protocol (for which we received IRB approval) as Supporting Information in our resubmission (S8 Study Protocol).
