# Supplementary material for: Impact evaluation of a digital health platform empowering Kenyan women across the pregnancy-postpartum care continuum: A cluster randomized controlled trial
Source: PLoS Med. 2025 Feb 3;22(2):e1004527. doi: 10.1371/journal.pmed.1004527 (PMC11835334; doi:10.1371/journal.pmed.1004527)
Supplement: Attachment — Submitted filename: PLOS Medicine Re-Revised Manuscript - Reviewer Response.docx [file pmed.1004527.s014.docx]

**Date:** 2 January 2025

**Manuscript/Revision Number:** PMEDICINE-D-24-01960R2

**Article Title:** “Impact evaluation of a digital health platform empowering Kenyan women across the pregnancy-postpartum care continuum: A cluster randomized controlled trial”

**Corresponding Author:** Rajet Vatsa ([rajet_vatsa@hms.harvard.edu](mailto:rajet_vatsa@hms.harvard.edu))

Dear *PLOS Medicine* Editorial and Review Team:

Thank you so much for your continued interest in our team’s research article. We appreciate the additional suggestions to strengthen our work for publication in your journal.

In our enclosed revision, we have attempted to address each of the comments provided and describe our changes in a point-by-point manner (see below). Page and line numbers refer to the manuscript version with no markup. Other general editorial comments that are not specifically addressed (e.g., abstract contents, use of abbreviations) have been reviewed in the manuscript and deemed to not require further action.

Again, thank you for all the feedback to help us get to this stage. Please do not hesitate to reach out if we can clarify any of our responses/revisions. We appreciate your continued time and consideration and look forward to hearing back.

Sincerely,

Rajet Vatsa, on behalf of the authorship team

Harvard University PhD Program in Health Policy | [Rajet_Vatsa@hms.harvard.edu](mailto:Rajet_Vatsa@hms.harvard.edu) | +1-480-747-4380

**Point-by-Point Response to Editorial and Reviewer Comments:**

General Editorial Requests

1. Please review your reference list to ensure that it is complete and correct… Any changes to the reference list should be mentioned in the rebuttal letter that accompanies your revised manuscript.

In reviewing our reference list, two related changes were made in the revised manuscript. Previously, we suggested in the Introduction (2^nd^ paragraph) that postpartum care in Kenya primarily focused on needs of the newborn over those of the mother. Support for that claim came from [this paper](https://pmc.ncbi.nlm.nih.gov/articles/PMC5691856/#CR8), from which we cited the study described by Family Care International (FCI). However, we can no longer find the underlying study online; it appears that FCI is no longer an active organization, and its website has been taken down. To be safe, we have removed the respective claim from our Introduction. Our Author Summary was the only other place that highlighted the overemphasis on newborn care over maternal care postnatally; we reframed the respective bullet (Lines 99-101) to focus on postpartum care more generally (for both mothers and newborns). While we had not previously cited the paper that contained the FCI reference, we have now added it (Line 132; Reference 21), as further evidence of the gaps in health information that pregnant and postpartum women experience.

2. Please confirm that your title complies with to *PLOS Medicine*’s style. Your title must be nondeclarative and not a question. It should begin with main concept if possible… Please place the study design… in the subtitle (i.e., after a colon).

We have revised our title so that it now complies with the journal’s style: “Impact evaluation of a digital health platform empowering Kenyan women across the pregnancy-postpartum care continuum: A cluster randomized controlled trial.”

3. The funding statement should include: specific grant numbers, initials of authors who received each award, URLs to sponsors’ websites… It appears that one or more study authors is affiliated with one or more of the agencies that funded the study… Please revise the Financial Disclosure accordingly.

Thank you for raising these issues. Upon further review with Jacaranda Health’s Finance Manager, we have updated our funding statement to include two additional sources of funding that were not previously listed. While the vast majority of the study was funded by the United States Agency for International Development (USAID)’s Development Innovation Ventures (DIV) program (previously reported), some funding support was also received from the Bill & Melinda Gates Foundation, as well as Jacaranda Health’s independent funds. The URLs to all sponsors’ websites are listed.

Regarding the comment that one or more study authors is affiliated with one or more of the agencies that funded the study, Jacaranda Health allocated limited, independent funds to the study. We have updated the Financial Disclosure to acknowledge this.

4. Please confirm that the appropriate usage rights apply to the use of this map.

We confirmed that the shapefile we used to generate the base map for Figure 2 is open-source and available under a Creative Commons Attribution license: see [this link](https://open.africa/dataset/kenya-counties-shapefile/resource/0b78f25e-494e-4258-96b8-a3ab2b35b121). In addition to including a direct link to the shapefile in the figure caption, we have added that the base map is “available under a Creative Commons Attribution license” (Lines 271-272).

5. The sample size listed in the submitted manuscript and the trial registry differ. Please explain the discrepancy.

This is an important point to clarify; thank you for raising it. In our manuscript, we mention an enrollment goal of 4,800 women (Line 406). However, in our record on the American Economic Association RCT Registry, we indicate a planned sample size of 10,000 individuals, including patients and providers at maternity facilities. The Study Protocol (S8) reconciles this. Recall that treatment facilities were exposed to two interventions: PROMPTS and MENTORS, Jacaranda Health’s complementary nurse mentorship program. Though we evaluate each intervention separately, the trial registry accounts for the sample size across both interventions. As described on Page 9 of the Study Protocol (S8), the goal was to enroll ~4,800 women for PROMPTS, ~4,800 women for MENTORS, and ~200 nurses and midwives for a provider assessment (more pertinent to MENTORS). Thus, the sample size listed in the manuscript (specific to PROMPTS) is consistent with the initial study protocol.

6. The CONSORT flowchart should be figure 1, please revise.

We have rearranged when we first introduce the map of facilities and CONSORT flowchart, such that the CONSORT flowchart is now Figure 1, while the facility map is Figure 2. Figure 1 is introduced on Line 205, while Figure 2 is introduced on Line 255.

Reviewer 3

7. On L117, antenatal care is abbreviated as ANC. The same was done on L353 and antenatal care is used several times without abbreviation (L156, for example). On L210, prenatal care appears but is not abbreviated as PNC.

Thank you for bringing this to our attention. We have made several changes to standardize things:

- We have replaced all instances of the world “prenatal” with the word “antenatal.”
- After the first instance of the phrase “antenatal care,” which we abbreviate as ANC (Line 115), we only use the abbreviated form, apart from the Figure 4 title and caption.
- After the first instance of the phrase “postnatal care,” which we abbreviate as PNC (Line 354), we only use the abbreviated form, apart from the Figure 4 title and caption.

8. In Tables 4 and 5, asterisks are used for p-value. Is it necessary? The P-value is written in the same tables.

Thank you for raising this point. We appreciate the comment but have ultimately elected to keep the table as is for the ease of readers who may be quickly skimming the table and/or are accustomed to looking for asterisks to denote significance. We defer to the *PLOS Medicine Editorial Team* for their final stance on the preferred formatting of our tables.

9. For questions 28,29 and 30, I understood the lack of data about ANC and PNC. Your response sounds reasonable, but I wonder if it should be included as a limitation as the timing of these cares is crucial.

Again, we appreciate this comment. We agree and have added two sentences to our Discussion acknowledging this limitation (Lines 741-744).
